# TOX2 coordinates with TET2 to positively regulate central memory differentiation in human CAR T cells

**DOI:** 10.1126/sciadv.adh2605

**Published:** 2023-07-19

**Authors:** Sierra M. Collins, Katherine A. Alexander, Stefan Lundh, Alexander J. Dimitri, Zhen Zhang, Charly R. Good, Joseph A. Fraietta, Shelley L. Berger

**Affiliations:** ^1^ Department of Cell and Developmental Biology, Perelman School of Medicine, University of Pennsylvania, Philadelphia, PA 19104, USA.; ^2^ Department of Pathology and Laboratory Medicine, Perelman School of Medicine, University of Pennsylvania, PA 19104, USA.; ^3^ Center for Cellular Immunotherapies, Perelman School of Medicine, University of Pennsylvania, Philadelphia, PA 19104, USA.; ^4^ Department of Microbiology, Perelman School of Medicine, University of Pennsylvania, Philadelphia PA 19104, USA.; ^5^ Abramson Cancer Center, Perelman School of Medicine, University of Pennsylvania, Philadelphia, PA 19104, USA.; ^6^ Epigenetics Institute, University of Pennsylvania, Philadelphia, PA 19104, USA.; ^7^ Department of Genetics, University of Pennsylvania, Philadelphia PA 19104, USA.; ^8^ Department of Biology, University of Pennsylvania, Philadelphia PA 19104, USA.

## Abstract

Chimeric antigen receptor (CAR) T cell therapy is used in treating human hematological malignancies, but its efficacy is limited by T cell exhaustion (T_EX_). T_EX_ arises at the expense of central memory T cells (T_CM_), which exhibit robust antitumor efficacy. Reduction of the TET2 gene led to increased T_CM_ differentiation in a patient with leukemia who experienced a complete remission. We show that loss of TET2 led to increased chromatin accessibility at exhaustion regulators TOX and TOX2, plus increased expression of TOX2. Knockdown of TOX increased the percentage of T_CM_. However, unexpectedly, knockdown of TOX2 decreased T_CM_ percentage and reduced proliferation. Consistently, a T_CM_ gene signature was reduced in the TOX2 knockdown, and TOX2 bound to promoters of numerous T_CM_ genes. Our results thus suggest a role for human TOX2, in contrast to exhaustion regulator TOX, as a potentiator of central memory differentiation of CAR T cells, with plausible utility in CAR T cell cancer therapy via modulated TOX2 expression.

## Teaser

TOX2 positively regulates central memory CAR T cell differentiation unlike the related exhaustion regulator TOX.

## INTRODUCTION

The cancer immunotherapy field has produced notable results that the adoptive transfer of genetically engineered T cells can mediate durable and complete remissions in patients with a variety of refractory hematologic cancers (*1*, *2*). Accordingly, chimeric antigen receptor (CAR) T cells have powerful antitumor effects in leukemia, lymphoma, and myeloma (*3*–*5*). However, despite this great clinical potential, in many patients, CAR T cells do not proliferate, durably persist, or elicit effective antitumor activity (*6*). A certain level of CAR T cell clonal expansion and persistence appears necessary for initial and durable benefit, but the mechanisms remain unclear. Poor persistence and loss of cytotoxicity, both indicators of dysfunction, are major determinants of resistance against CAR T cells (*6*). One important finding is that T cell exhaustion often occurs at the expense of differentiation into stem-like or central memory cells, which have superior antitumor activity and prolonged persistence in preclinical CAR T cell models and in patients treated with CAR T cells (*7*). These findings suggest that modulation of transcriptional regulators to enforce T cell memory–associated programs will increase persistence and antitumor efficacy.

One factor that regulates CAR T cell function is the DNA demethylase Tet methylcytosine dioxygenase 2 (TET2) (*7*). We profiled a patient with chronic lymphocytic leukemia (CLL) who had a complete and durable response to CAR T cell therapy and found that the CAR construct (which is inserted into the genome by a lentivirus) had disrupted the *TET2* locus, rendering it nonfunctional. The patient had a missense mutation in the second allele of *TET2* that compromised its demethylase activity, culminating in biallelic disruption of the gene. Postinfusion sequencing revealed that CAR T cells with this *TET2* disruption were positively selected, had undergone a large clonal expansion, and led to a higher CAR T cell proportion differentiating into central memory T cells (T_CM_), a memory subset that persist for long periods of time in the body and have greater self-renewal potential than other later-stage memory subtypes. In vitro knockdown proved that reduction of *TET2* was sufficient to increase the frequency of T_CM_ and to improve proliferation in response to antigen-presenting cells, which is a common measure of CAR T cell antitumor efficacy (*7*).

However, while the disruption of *TET2* improves CAR T cell therapy, its use as a therapeutic strategy may be limited because TET2 is a tumor suppressor. Thus, there may be detrimental long-term effects of reducing TET2 function in human CAR T cells, as it may safeguard against aberrant lymphoproliferation. Alternative strategies that target specific factors without broad functions like TET2 may be more beneficial for CAR T therapy.

Previous studies reveal up-regulation of thetranscription factor Thymocyte selection-associated high mobility group box protein (TOX) in T cell exhaustion with a crucial role in promoting T cell exhaustion (*8*). Mouse models of CAR T therapy also show increased expression of *TOX* in tumor-infiltrating lymphocytes (TILs), as well as the closely related transcription factor, *TOX2* (*9*). Mouse models of chronic infection show up-regulation of both *TOX* and *TOX2* in exhausted T cells, with TOX playing a direct role in regulating transcription of *TOX2*. Simultaneous loss of *TOX* and *TOX2* improves the antitumor potency of TILs, but loss of *TOX2* alone is not sufficient to significantly reduce tumor burden, suggesting that, in mouse models, the contribution of TOX2 to exhaustion is weaker compared to TOX (*9*).

Outside T cell exhaustion, TOX2 can improve function in other cellular contexts. TOX2 is required for proper maturation of cytotoxic human natural killer (NK) cells, which are positively regulated by TOX2, inducing expression of the gene *TBX21* (*10*). TOX2 also up-regulates *Tbx21* in mouse type I invariant NK T (iNKT1) cells; defects in iNKT1 cells are associated with diseases such as rheumatoid arthritis (*11*). In addition, TOX2 is a key regulator of the differentiation of mouse T follicular helper (Tfh) cells. In Tfh cells, TOX2 has been consistently found to improve function by suppressing inflammation-related gene pathways (*12*). Thus, the precise function of TOX2 appears to be highly cell context and T cell type dependent.

CAR T cell therapy is typically administered as a mixed population of CD4^+^ and CD8^+^ T cells, conventionally considered helper cells and cytotoxic cells, respectively. They are distinct subsets with different functional and transcriptional characteristics, including variations in ability to proliferate and persist in vivo following in vitro expansion and adoptive transfer (*2*). Previous studies of TET2 and other factors that affect CAR T cell cytotoxicity and persistence have mainly focused on CD8^+^ T cells (*7*). However, we recently found that CAR T–treated patients with CLL have a preponderance of clonally expanded CD4^+^ CAR T cells with cytotoxic function that correlates with long-term durable remission (*2*). This highlights the importance of considering both CD4^+^ and CD8^+^ T cells in the context of CAR T cell therapy, as both subsets contribute to the overall treatment efficacy.

In this study, we aimed to gain a deeper understanding of TOX compared to TOX2 in human CD4^+^ and CD8^+^ CAR T cells, prompted by our observation of chromatin opening at both genes in the patient with CLL with disrupted *TET2*. Our results show that TOX2 plays a critical role in the development of T cell memory by increasing T_CM_ differentiation, while, in contrast, TOX promotes T cell exhaustion by reducing the generation of T_CM_. These findings have important implications for potential human CAR T therapy.

## RESULTS

### *TET2* loss promotes *TOX* and *TOX2* chromatin accessibility

**Fig. 1.**
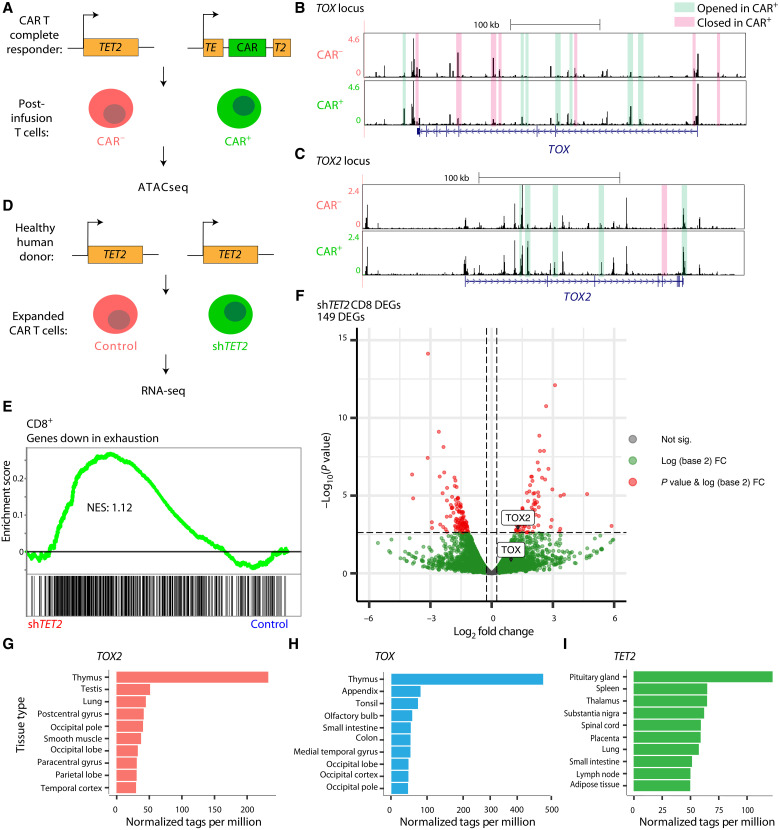
*TET2 *loss induces chromatin changes at *TOX* and *TOX2*. (**A**) Our prior study (*7*) examined the chromatin accessibility of postinfusion T cells from a patient (*n* = 1) who responded completely to chimeric antigen receptor (CAR) T therapy using assay for transposase accessible-chromatin using sequencing (ATACseq) to compare CAR^+^ to CAR^−^ cells. This patient had biallelic disruption of *TET2*. (**B**) ATACseq browser tracks for CAR^−^ (*n* = 2 technical replicates) and CAR^+^ (*n* = 2 technical replicates), shaded boxes indicate MACS2 (*28*) called peaks with log_2_fold change > 1 (green) or <−1 (pink), tracks visualized through http://genome.ucsc.edu, showing the TOX locus and (**C**) TOX2 locus. (**D**) Schematic of RNA-seq comparing healthy human donor T cells treated with nontargeting short hairpin RNA (shRNA) as a control (*n* = 2) versus shRNA targeting *TET2* (*n* = 2). (**E**) Volcano plot showing log_2_fold change versus −log_10_(*P* value) of all genes in CD8^+^ CAR^+^ T cells for sh*TET2* versus control, with *P*_adj_ < 0.18 and fold change > 0.25 [calculated using DESeq (*43*)] considered significant. (**F**) Gene set enrichment analysis (GSEA) in CD8^+^ sh*TET2* (*n* = 2) versus control (*n* = 2) T cell counts of a gene set down-regulated in exhausted T cells, normalized enrichment score (NES) = 1.12, false discovery rate (FDR) *q* = 0.05. (**G**) RNA-seq normalized tags per million from the FANTOM dataset showing expression in various tissues and organs of *TOX2*, (**H**) *TOX,* and (**I**) *TET2*.

To investigate TOX and TOX2 in human CAR T cell fate and function, we first analyzed the regulation of these genes in a CAR T cell context with robust antitumor patient response. Our previous study identified biallelic disruption of the *TET2* gene in expanded CAR T cells from a patient who had responded completely to CAR T therapy (*7*). Furthermore, we found that in vitro loss of *TET2* is sufficient to increase the frequency of T_CM_ cells and to improve proliferation in response to antigen (*7*). Using available assay for transposase accessible-chromatin using sequencing (ATACseq) data generated from our study of these patient-derived *TET2*-disrupted postinfusion CD8^+^ CD19 CAR T cells, we compared the *TOX* and *TOX2* loci to a negative control of patient-matched CD8^+^ T cells that did not express the CAR (Fig. 1A).

We found that the *TOX* locus included seven ATACseq peaks with a log_2_–fold change greater than one (Fig. 1B) and the *TOX2* locus included five such peaks (Fig. 1C). The chromatin opening of *TOX* and *TOX2* in these CAR T cells from a robustly responding patient is unexpected because they are both linked to T cell exhaustion in mouse. To examine the transcription state, we used knockdown of *TET2* in donor human CAR T cells (Fig. 1D) to recapitulate the loss of *TET2* observed in the patient. Using gene set enrichment analysis (GSEA), we checked for enrichment in the *TET2* knockdown versus the control groups of gene sets associated with T cell exhaustion (*13*) to determine whether the increased chromatin accessibility at *TOX* and *TOX2* in the patient data correlated with an increase in T cell exhaustion in the donor human CAR T cells. We found that the RNA sequencing (RNA-seq) data following sh*TET2* treatment was significantly enriched for a gene set that is down-regulated in T cell exhaustion (Fig. 1E), suggesting that *TET2* loss does not promote exhaustion. Upon examining transcriptional targets of *TET2* loss, we found that *TOX2* ranked 71st on the list of most significantly up-regulated genes in the sh*TET2*, whereas *TOX* was not significantly up-regulated (Fig. 1F). We more closely examined the locations of the ATACseq peaks from the patient and found that two of the opened peaks at the *TOX2* locus overlapped with enhancers previously found to interact with the promoter of *TOX2* (*14*), potentially explaining the increased *TOX2* transcription. In contrast, an enhancer that normally interacts with the promoter of *TOX* (*14*) was closed in the CAR T cells, suggesting decreased transcription. Together, this suggests that *TOX2* is up-regulated by loss of *TET2*, and it highlights that *TOX2* can be up-regulated separately from *TOX* in non–T cell exhaustion settings.

Considering concerns about the potential lack of specificity when directly manipulating *TET2* in CAR T cells and TET2’s role in cancer, we compared the tissue-specific expression levels of *TET2*, *TOX*, and *TOX2*. Using published data (*15*–*17*), we found that the expression of *TOX* and *TOX2* is at least fourfold higher in the thymus than in any other tissue type (Fig. 1, G and H), underscoring their role in T cell biology. However, *TET2* is widely expressed throughout the body (Fig. 1I). Thus, although *TET2* loss has been clinically beneficial (*7*), *TOX2* is a potentially superior target for manipulation in CAR T cells because of its specificity within the T cell compartment and increased chromatin accessibility in a patient who responded completely to CAR T therapy.

### *TOX2* is necessary for T_CM_ differentiation

**Fig. 2.**
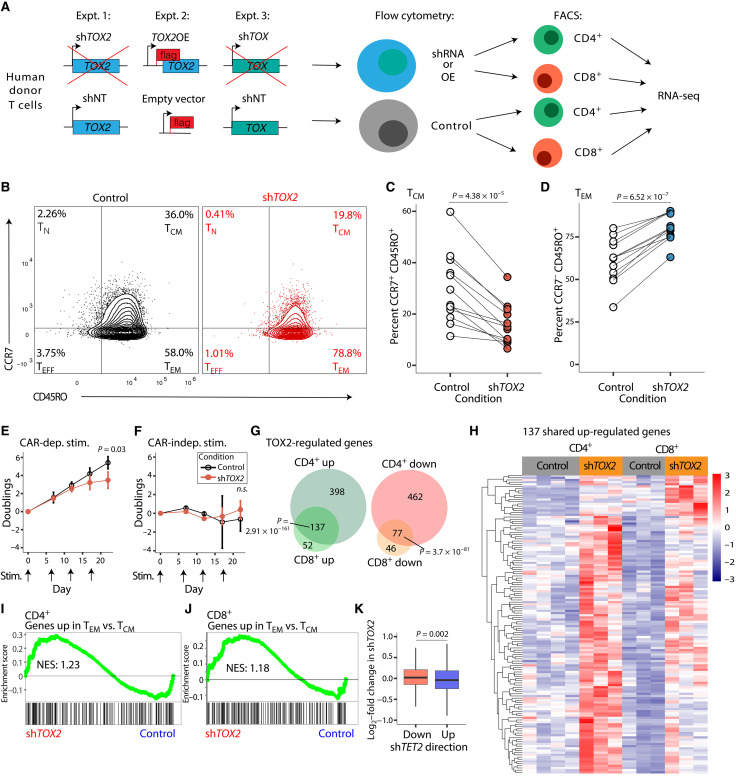
*TOX2 *is necessary for maximal central memory T cell (T_CM_) formation. (**A**) Schematic of experimental design. (**B**) Representative flow cytometry (*n* = 1) comparing day 14 in vitro–expanded human CAR19 T cells treated with nontargeting control short hairpin RNA (shRNA) versus shRNA targeting *TOX2*, plotted as CD45RO versus CCR7. (**C**) Percentage of CD45RO^+^CCR7^+^CAR19^+^ T cells in seven human donors over four separate experiments, 12 samples in total, paired Student’s *t* test, *P* = 4.38 × 10^−5^. (**D**) Percentage of CD45RO^+^CCR7^−^CAR19^+^ T cells in seven human donors over four separate experiments, 12 samples in total, paired Student’s *t* test, P = 6.54 × 10^−7^. (**E**) Population doublings after 7, 12, 17, and 22 days for control versus sh*TOX2* CAR19^+^ T cells cocultured with CD19-presenting K562 cells (*n* = 3, paired Student’s *t* test, *P* = 0.03). (**F**) Population doublings after 7, 12, 17, and 22 days for control versus sh*TOX2* CAR19^+^ T cells cocultured with mesothelin-presenting K562 cells (*n* = 3, paired Student’s *t* test, *P* > 0.05, n.s., not significant). (**G**) Numbers of genes up-regulated in sh*TOX2* (*n* = 4) versus control (*n* = 4) in both CD4^+^ T cells and CD8^+^ T cells, plus genes down-regulated in sh*TOX2* versus control in both CD4^+^ T cells and CD8^+^ T cells. Hypergeometric test, *P* = 2.91 × 10^−161^ and *P* = 3.7 × 10^−81^. (**H**) Heatmap of *z* scores from normalized RNA-seq counts from the 137 genes up-regulated in both CD4^+^ and CD8^+^ sh*TOX2* versus control, clustered by row. (**I**) Gene set enrichment analysis (GSEA) in CD4^+^ sh*TOX2* versus control T cell counts of a gene set up-regulated in effector memory T cells (T_EM_) versus T_CM_, NES = 1.23, FDR *q* = 0.09. (**J**) GSEA in CD8^+^ sh*TOX2* versus control T cell counts of a gene set up-regulated in effector memory cells versus T_CM_, normalized enrichment score (NES) = 1.18, false discovery rate (FDR) *q* = 0.13. (**K**) Sets of genes down-regulated or up-regulated in the sh*TET2* versus control from Fig. 1D, plotted as box plots of their log_2_–fold change in the sh*TOX2* versus control, unpaired Student’s *t* test, *P* = 0.002.

To address the specific roles of TOX and TOX2 in CAR T cells, we altered their expression. We hypothesized that modulation of *TOX* and *TOX2* levels would affect the memory differentiation of CAR T cells, similar to loss of *TET2* (*7*). To test this, we used short hairpin RNA (shRNA) knockdown of *TOX2* in human CAR T cells and examined their immunophenotype (Fig. 2A, Expt. 1). We used a previously established T cell differentiation panel that identifies memory subtypes based on the expression of cell surface markers CCR7 (high in T_CM_) and CD45RO [high in both T_CM_ and effector memory T cells (T_EM_)] (*7*). We found that loss of *TOX2* significantly reduced CD45RO^+^CCR7^+^ T_CM_ cells (Fig. 2B, top right; quantified in Fig. 2C and table S1), while it significantly increased the CD45RO^+^CCR7^−^ (T_EM_) population (Fig. 2B, bottom right; quantified in Fig. 2D and table S1). We also validated this using a second marker, CD27, which showed decreased T_CM_ cells via a drop in the CD45RO^+^ CD27^+^ population (fig. S1A and table S2). These data indicate that TOX2 may regulate human CAR T cell differentiation via enriching T_CM_ cells, which have robust antitumor potency (*18*).

To investigate whether the reduction of TOX2 that impairs T_CM_ cell outgrowth can decrease the proliferative potency of CAR T cells, we performed a serial stimulation experiment using CAR T cells and irradiated K562 chronic myeloid leukemia (CML) cells engineered to express the CD19 extracellular domain. This “stress test” assay closely mimics features of antigen-induced exhaustion during long-term coculture (*19*). The restimulation assay accurately predicts the proliferative potency of CAR T cells in patients, which has a direct and strong correlation with clinical outcome (*20*, *21*). We found, after 22 days of chronic antigen challenge, that cells containing the sh*TOX2* had significantly fewer cell divisions than CAR T cells with a scrambled control shRNA (Fig. 2E and table S3). To confirm that this reduction in cell number was antigen dependent, we cocultured the manipulated CAR T cells with mesothelin-expressing K562 cells as an irrelevant control, which do not interact with the CD19-targeting CAR. There was no difference in cell number between the control and sh*TOX2* (Fig. 2F and table S3), indicating that reduced proliferation caused by *TOX2* loss was antigen dependent. These results demonstrate that TOX2 is necessary for maximal CAR T cell growth in the presence of antigen.

Similar to CAR T therapy, our coculture experiment used a mixed population of CD4^+^ and CD8^+^ T cells. To investigate whether *TOX2* knockdown affected CD4^+^ cells differently from CD8^+^ T cells, we performed RNA-seq on sorted CD4^+^ and CD8^+^ CAR T cells. Levels of *TOX2* were significantly reduced in both CD4^+^ and CD8^+^ sh*TOX*2 samples compared to controls, although we noted that endogenous levels of *TOX2* were higher in CD4^+^ cells, and the reduction in *TOX2* with the shRNA was larger (fig. S1B). We identified 1074 changing genes in CD4^+^ T cells (fig. S1C) but only 312 in CD8^+^ T cells (fig. S1D). A majority of differentially expressed genes in CD8^+^ T cells were also changing in CD4^+^ T cells (68.5%), whereas the CD4^+^ T cells had many more differentially expressed genes that were unaltered in CD8^+^ T cells (Fig. 2G). These results suggest that *TOX2* loss had a greater effect on CD4^+^ T cells in our experimental system. Even for the 137 genes up-regulated in both CD4^+^ T cells and CD8^+^ T cells with the *TOX2* knockdown, a heatmap showed a trend toward stronger, more consistent up-regulation in CD4^+^ T cells (Fig. 2H). Although the average fold change of the *TOX2* knockdown over the control for these genes was higher in CD8^+^ than CD4^+^ cells (fig. S1E), the normalized expression levels of the genes were higher in CD4^+^ cells (fig. S1F). These findings indicate that, in addition to regulating more genes in CD4^+^ CAR T cells, *TOX2* loss leads to higher expression of individual genes.

Using GSEA, we checked for enrichment in the *TOX2* knockdown versus the control groups of gene sets associated with various T cell states (table S4) (*22*). We found that a previously described set of genes up-regulated in T_EM_ compared to T_CM_ (*22*) were significantly enriched in the sh*TOX2* in both CD4^+^ cells (Fig. 2I) and CD8^+^ cells (Fig. 2J). The reverse was also true in CD4^+^ cells, in that genes down-regulated in T_EM_ compared to T_CM_ (*22*) cells were enriched in the control sample (table S4). These results indicate that loss of *TOX2* drives progressive, antigen-dependent CAR T cell differentiation from the T_CM_ to T_EM_ state, particularly in CD4^+^ T cells. In addition, we speculate that TOX2 may promote long-term maintenance of CD4^+^ T cells, as they can exhibit longer persistence in CAR T cell therapy (*2*).

To further predict the consequences of *TOX2* loss on clinical CAR T therapy, we compared transcriptional gene targets of *TOX2* loss to those of *TET2* loss. We examined mRNA levels from the sh*TET2* in Fig. 1. We grouped all genes either into down-regulated (*N* = 7000) or up-regulated (*N* = 7007) and then plotted as log_2_–fold changes in our sh*TOX2* cells (Fig. 2K). We found that the genes up-regulated in the *TET2* loss had, on average, a significantly more negative fold change in the *TOX2* loss than the genes down-regulated in *TET2* loss (*P* = 0.002; Fig. 2K). These results are consistent with the interpretation that some of the positive gene expression gained in *TET2* loss would be eliminated upon *TOX2* loss, and thus, certain positive effects of losing *TET2* may be dependent on the presence of *TOX2*.

### TOX2 is sufficient to increase central memory differentiation but not to improve CAR T cell function

**Fig. 3.**
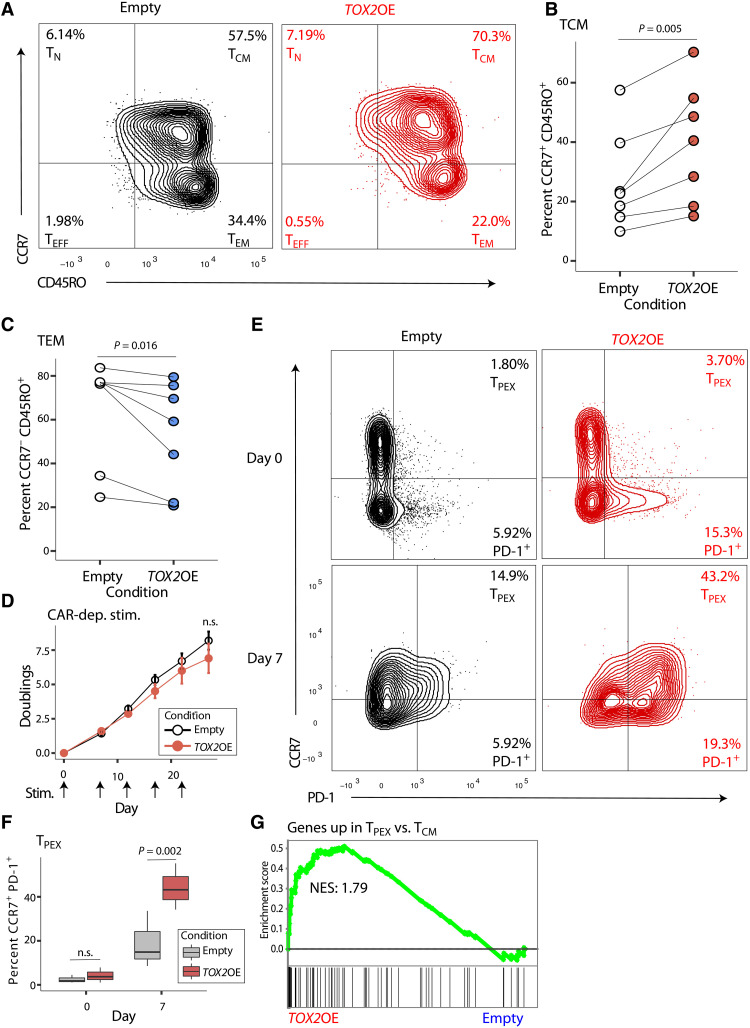
TOX2 is sufficient to increase central memory phenotype but not to improve chimeric antigen receptor (CAR) T cell function. (**A**) Representative flow cytometry (*n* = 1) comparing day 14 in vitro–expanded human CAR19 T cells treated with empty control virus versus *TOX2* cDNA virus, plotted as CD45RO versus CCR7. (**B**) Percentage of CD45RO^+^CCR7^+^CAR19^+^ T cells in five human donors over four separate experiments, seven samples total, paired Student’s *t* test, *P* = 0.005. (**C**) Percentage of CD45RO^+^CCR7^−^ CAR19^+^ T cells in five human donors over four separate experiments, seven samples total, paired Student’s *t* test, *P* = 0.016. (**D**) Population doublings after 7, 12, 17, 22, and 27 days for control versus *TOX2*OE CAR19^+^ T cells cocultured with CD19-presenting K562 cells (*n* = 3, paired Student’s *t* test, *P* > 0.05; n.s., not significant). (**E**) Representative flow cytometry (*n* = 1) comparing day 0 and day 7 control versus *TOX2*OE CAR19^+^ T cells from (D), plotted as PD-1 versus CCR7. (**F**) Percentage of PD-1^+^CCR7^+^CAR19^+^ T cells from three human donors at days 0 and 7, control (*n* = 3) versus *TOX2*OE (*n* = 3), paired Student’s *t* test, *P* > 0.05 and *P* = 0.002. (**G**) Gene set enrichment analysis (GSEA) in CD8^+^ *TOX2*OE versus control (*n* = 3) RNA-seq normalized counts of a gene set up-regulated in T cell exhaustion–precursor (T_PEX_) cells, normalized enrichment score (NES) = 1.79, false discovery rate (FDR) *q* = 0.001.

Thus, loss of *TOX2* led to reduction of T_CM_. We hypothesized that, if TOX2 is sufficient to drive the T_CM_ phenotype, increasing the levels of *TOX2* would shift differentiation toward the T_CM_ state and lead to improvement in CAR T cell function. To test this, we overexpressed *TOX2*-FLAG in human CAR T cells (Fig. 2A, Expt. 2). We used flow cytometry as described above to separate memory subtypes based on CD45RO and CCR7 expression. *TOX2* overexpression significantly increased the proportion of CD45RO^+^CCR7^+^ T_CM_ (top right quadrant in Fig. 3A; quantified in Fig. 3B and table S5) and correspondingly decreased the proportion of CD45RO^+^CCR7^−^ T_EM_ (bottom right quadrant of Fig. 3A; quantified in Fig. 3C and table S5). It also trended toward increasing the proportion of CD45RO^+^CD27^+^ T_CM_ (fig. S2A and table S2). These results demonstrate that elevated expression of *TOX2* alters the differentiation path of human CAR T cells in favor of a longer-lasting, central memory phenotype.

In previous studies, an outgrowth of CAR T_CM_ cells after infusion correlates with improvement in CAR T cell function in patients (*7*). Therefore, we tested whether increasing *TOX2* levels would improve CAR T cell proliferation in response to antigen. The CAR^+^ T cells were sorted and then cocultured for 27 days with the CD19-presenting K562 cell line. Unexpectedly, there was no change in cell number doublings between the empty vector and the *TOX2* overexpression (Fig. 3D and table S6). Hence, while TOX2 is necessary for optimal CAR T cell proliferation (Fig. 2) and *TOX2* overexpression increases T_CM_ differentiation (Fig. 3, A to C), *TOX2* overexpression is not sufficient to increase growth above the level of the control.

Next, we investigated the impact of *TOX2* overexpression on CAR T cell function and growth because our observations above showed that *TOX2* overexpression increased the frequency of CAR T_CM_ cells but did not increase cell proliferation. We performed flow cytometry after 7 days of coculture and found that the percentage of programmed cell death protein 1 (PD-1)^+^ cells was significantly increased in the group with *TOX2* overexpression [Fig. 3E (bottom right quadrant); quantified in fig. S2B and table S7]. Many of these PD-1^+^ cells were also CCR7^+^ (Fig. 3E; quantified in Fig. 3F and table S7). This population of cells, known as T cell exhaustion–precursor cells (T_PEX_), complicates traditional distinctions between memory T cell subsets and shows reduced proliferation and cytokine secretion compared to other T cell types (*23*). Thus, our results suggest that *TOX2* overexpression promotes T_PEX_ differentiation, which may explain the lack of improvement in CAR T cell function despite an increase in beneficial T_CM_.

Using RNA-seq, we further investigated whether T_PEX_ cells develop in the *TOX2* overexpression state. GSEA revealed that a set of genes up-regulated in T_PEX_ compared to T_CM_ (*23*) was enriched with *TOX2* overexpression (Fig. 3G). Exhaustion-related genes were increased, such as *PDCD1* (encoding PD-1), *NKG7*, and *GZMK*, and these were also up-regulated in T_PEX_ (fig. S2C, top). In addition, *TOX2* overexpression down-regulated many genes (including *IL6ST*, *MYC*, *DPP4*, and *SATB1*) that are also down-regulated in T_PEX_ (fig. S2C, bottom). Hence, *TOX2* overexpression leads not only to increased T_CM_ signature but also to increased T_PEX_ signature [as in (*23*)], which likely explains the limited overall improvement in T cell function.

### TOX2 promotes both central memory and exhaustion gene signatures

Many studies of exhaustion focus on CD8^+^ T cells because the loss of CD8^+^ cytotoxicity is thought to underlie the negative effects of exhaustion in contexts like CAR T therapy. However, as mentioned above, evidence suggests that clonal expansion of CD4^+^ CAR T cells is linked to long-term remission in patients (*2*). While our immunophenotyping assays assessed a combined population of CD4^+^ and CD8^+^ cells, we can examine these cells separately through RNA-seq. We thus sorted the *TOX2* overexpressing CAR^+^ cells into CD4^+^ and CD8^+^ subgroups, performed RNA-seq, and confirmed that *TOX2* levels were greatly increased in both CD4^+^ and CD8^+^ cells (fig. S2D). We identified 655 changing genes in CD4^+^ cells (fig. S2E) and 404 changing genes in CD8^+^ cells (fig. S2F), indicating that overexpressed *TOX2* regulates more genes in CD4^+^ than CD8^+^ cells, consistent with our sh*TOX2* results (Fig. 2G). We found 95 genes down-regulated by *TOX2* overexpression in both CD4^+^ and CD8^+^ cells. In CD4^+^ cells, the mean log_2_–fold change of these 95 genes was −1.21, compared to −0.92 in CD8^+^ cells (*P* = 1.43 × 10^−7^; fig. S2G), showing that *TOX2* overexpression leads to larger changes in average gene expression in CD4^+^ cells.

Having detected the presence in *TOX2* overexpression of increased signatures of both T_CM_ and T_PEX_, we then examined whether there was transcriptional specificity between CD4^+^ and CD8^+^ cells for the two cell types. We performed the same GSEA as in Fig. 2. In CD4^+^ cells, but not CD8^+^, the control sample was enriched for genes up-regulated in T_EM_ versus T_CM_ (*22*), suggesting that the beneficial T_CM_ memory function of TOX2 may be stronger in CD4^+^ T cells. Note that, at baseline, *TOX2* was more highly expressed in CD4^+^ T cells compared to CD8^+^ (fig. S1A). In addition, there were consistently a greater number of differentially expressed genes in CD4^+^ T cells compared to CD8^+^ (figs. S1, B and C, and S2, D and E). In CD8^+^ cells, but not CD4^+^, the control samples were enriched for genes down-regulated in exhaustion pathways (table S8) (*13*). Thus, the CD4^+^ T cells may be contributing more strongly to memory function, whereas the CD8^+^ T cells may be contributing more to the T_PEX_ phenotype.

**Fig. 4.**
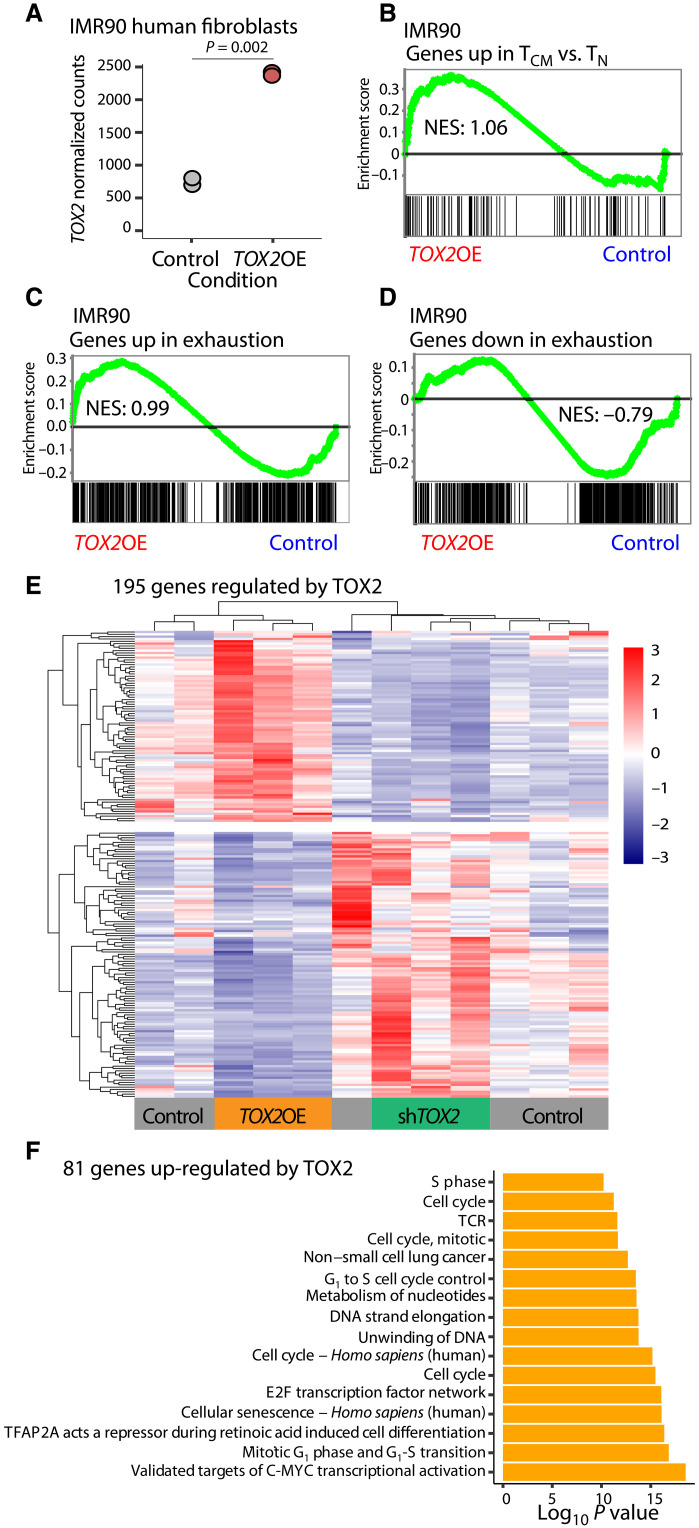
TOX2 promotes both central memory and exhaustion gene signatures. (**A**) Normalized RNA-seq counts of TOX2 in overexpression of FLAG-TOX2 (*n* = 2) versus expression of FLAG-empty (*n* = 2) in human IMR90 fibroblasts. (**B**) Gene set enrichment analysis (GSEA) in IMR90 *TOX2*OE (*n* = 3) versus control RNA-seq (*n* = 3) normalized counts of a gene set up-regulated in central memory T cells (T_CM_) versus naive T cells (T_N_), normalized enrichment score (NES) = 1.06, false discovery rate (FDR) *q* = 0.76. (**C**) GSEA in IMR90 *TOX2*OE (*n* = 3) versus control (*n* = 3) RNA-seq normalized counts of a gene set up-regulated in exhausted T cells (T_EX_), NES = 0.99, FDR *q* = 0.56. (**D**) GSEA in IMR90 *TOX2*OE (*n* = 3) versus control (*n* = 3) RNA-seq normalized counts of a gene set down-regulated in T_EX_, NES = −0.79, FDR *q* = 0.89. (**E**) Heatmap of *z* scores from normalized RNA-seq counts from the 195 genes changing in the *TOX2*OE (*n* = 3) and sh*TOX2* (*n* = 4) compared to their respective controls, clustered by row and column. (**F**) Pathway enrichment analysis by ConsensusPathDB for the 81 genes up-regulated by *TOX2*OE.

We then overexpressed *TOX2* in a non–T cell context to investigate whether TOX2 can invoke T_CM_ and T_EX_ gene signatures in a completely unrelated cell context, as activation in an unrelated system is a persuasive way to show that a factor has a dominant transcriptional function. We constitutively expressed FLAG-tagged TOX2 in the diploid human IMR90 lung fibroblast cell line. RNA-seq showed strong expression of *TOX2* (Fig. 4A). GSEA showed enrichment for genes associated with T_CM_ over naive (T_N_) cells (*24*) with *TOX2* overexpression (Fig. 4B), consistent with *TOX2* overexpression in T cells increasing the percentage of T_CM_ (Fig. 3B). Furthermore, we observed that the *TOX2* overexpression samples were also enriched for a gene set up-regulated in T_EX_ (Fig. 4C) (*13*), and the control samples were enriched for a gene set down-regulated in T_EX_ (Fig. 4D) (*13*). These results support our assertion above that TOX2 may regulate the intermediate phenotype T_PEX_.

### TOX2 regulates cell cycle genes

Thus, TOX2 may positively regulate memory gene signatures in CD4^+^ T cells. To reveal the most important genetic targets that may underlie the positive effects on memory, we combined the RNA-seq data from the *TOX2* overexpression in Fig. 3 with data from the sh*TOX*2 in Fig. 2. We focused on 81 genes up-regulated by *TOX2* overexpression and down-regulated by sh*TOX2*, as genes activated by TOX2 [Fig. 4E (top half) and table S9], and 114 genes down-regulated by *TOX2* overexpression and up-regulated by sh*TOX2*, as genes repressed by TOX2 [Fig. 4E (bottom half) and table S9]. Pathway analysis revealed that the genes activated by TOX2 are primarily enriched for cell cycle pathways (Fig. 4F), suggesting that TOX2 functions in cell growth and is consistent with the necessity of TOX2 for maximal CAR T cell proliferation in response to antigen (Fig. 2E). The genes repressed by TOX2 were not significantly enriched for any pathways of obvious interest. Together, we conclude that, while knockdown of *TOX2* shows that it is necessary for CAR T cell proliferation in response to antigen, overexpression of *TOX2* leads to simultaneous promotion of the T_PEX_ state, thus preventing improvement in proliferation.

### TOX is necessary for T_EM_ differentiation

**Fig. 5.**
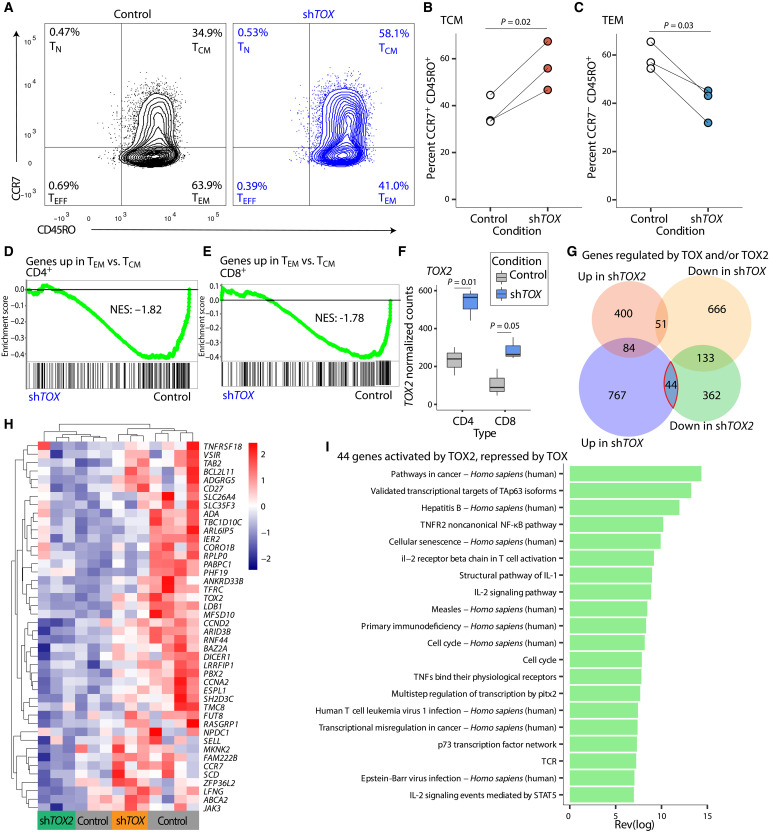
TOX is necessary for effector memory T cells (T_EM_) differentiation. (**A**) Representative flow cytometry (*n* = 1) comparing day 14 in vitro–expanded human CAR19 T cells treated with nontargeting control short hairpin RNA (shRNA) versus shRNA targeting *TOX*, plotted as CD45RO versus CCR7. (**B**) Percentage of CD45RO^+^CCR7^+^CAR19^+^ T cells in three human donors, paired Student’s *t* test, *P* = 0.02. (**C**) Percentage of CD45RO^+^CCR7^−^CAR19^+^ T cells in three human donors, paired Student’s *t* test, *P* = 0.03. (**D**) Gene set enrichment analysis (GSEA) in CD4^+^ sh*TOX* (*n* = 3) versus control (*n* = 3) T cell counts of a gene set up-regulated in T_EM_ versus central memory T cells (T_CM_), normalized enrichment score (NES) = −1.82, false discovery rate (FDR) *q* = 0. (**E**) GSEA in CD8^+^ sh*TOX* versus control T cell counts of a gene set up-regulated in T_EM_ versus T_CM_, NES = −1.78, FDR *q* = 0. (**F**) Normalized RNA-seq counts for *TOX2* in CD4^+^ and CD8^+^ CAR19^+^ T cells, control (*n* = 3) versus sh*TOX* (*n* = 3). (**G**) Overlap of genes up- or down-regulated in the sh*TOX* versus sh*TOX2* CD4^+^ T cells. (**H**) Heatmap of *z* scores from normalized RNA-seq counts from the 44 genes up-regulated in sh*TOX* and down-regulated in sh*TOX2*, clustered by row and column. (**I**) Pathway enrichment analysis by ConsensusPathDB for the 44 genes shown in (H).

Similar to the *TOX2* gene locus, the *TOX* locus exhibited many ATACseq peaks of increased chromatin accessibility in the patient who responded to CAR T therapy (Fig. 1B) (*7*). We therefore directly compared the effects of TOX and TOX2 on memory differentiation in CAR T cells (Fig. 2A, Expt. 3). We knocked down *TOX* in human CAR T cells and examined the effect on T cell memory differentiation (Fig. 5A). In contrast to the effects of knockdown of *TOX2*, which lowered T_CM_, knockdown of *TOX* led to increased percentages of CD45RO^+^CCR7^+^ T_CM_ (Fig. 5A, top right quadrant; quantified in Fig. 5B and table S10) and decreased frequencies of CD45RO^+^CCR7^−^ T_EM_ (Fig. 5A, bottom right quadrant; quantified in Fig. 5C and table S10). In addition, knockdown of *TOX* led to increased percentages of CD45RO^+^CD27^+^ T_CM_ in some donors (fig. S3A and table S2). This demonstrates that TOX plays a key role in differentiation of T_EM_, in contrast to our observations with TOX2, which enforces the central memory state at the expense of T_EM_ differentiation.

We performed RNA-seq on CD4^+^ and CD8^+^ T cells separately in the sh*TOX* and found that the levels of *TOX* decreased in both cell populations (fig. S3B). GSEA revealed that the gene set associated with increased T_EM_ compared to T_CM_ (*22*) was enriched in the control samples of both CD4^+^ (Fig. 5D) and CD8^+^ T cells (Fig. 5E), suggesting that TOX promoted differentiation of T_EM_ cells compared to T_CM_ cells. Previous work has suggested that a higher proportion of T_EM_ cells compared to T_CM_ cells leads to less effective tumor clearance during CAR T therapy (*25*), so these results are consistent with the idea that TOX is a driver of exhaustion in T cells, which also negatively affects the function of CAR T cells. However, our findings are not consistent with previous studies in mouse models, which suggest that TOX functions in a similar manner as TOX2 (*9*).

We observed that *TOX2* was significantly up-regulated upon knockdown of *TOX* (Fig. 5F), which could, in part, underlie increases in T_CM_ observed with knockdown of *TOX*. The up-regulation was stronger in CD4^+^ cells compared to CD8^+^ cells (Fig. 5F), suggesting that TOX may repress *TOX2* specifically in CD4^+^ T cells. Overall, loss of *TOX* had a stronger effect on CD4^+^ T cells, similar to TOX2: A total of 1745 genes changed in CD4^+^ sh*TOX* cells (fig. S3C), whereas 715 genes changed in CD8^+^ sh*TOX* cells (fig. S3D).

We compared the RNA-seq results from *TOX* knockdown versus *TOX2* knockdown and found that the majority of genes were uniquely regulated by either TOX or TOX2 (Fig. 5G). We were most interested in 44 genes that were down-regulated by *TOX2* knockdown and up-regulated by *TOX* knockdown (Fig. 5G, outlined in red); that is, given that T_CM_ are increased by gain of *TOX2* (Fig. 3B) and increased by loss of *TOX* (Fig. 5B), this core set of 44 genes (Fig. 5H) could be key to positive regulation of T_CM_. These genes were significantly enriched for interleukin-2 (IL-2) signaling pathways through the regulation of *JAK3* and *CCND2* (Fig. 5I). Recent studies show that IL-2 signaling can be harnessed to improve CAR T cell expansion, activation, and antitumor activity (*26*, *27*). Furthermore, in our experiments, negative effects on CAR T function of loss of *TOX2* could be due to loss in IL-2 signaling, which does not occur upon loss of *TOX*. Overall, we conclude that TOX and TOX2 provide differential gene regulation leading to different functional consequences in human CAR T cells.

### TOX2 binds to DNA at memory-related genes

**Fig. 6.**
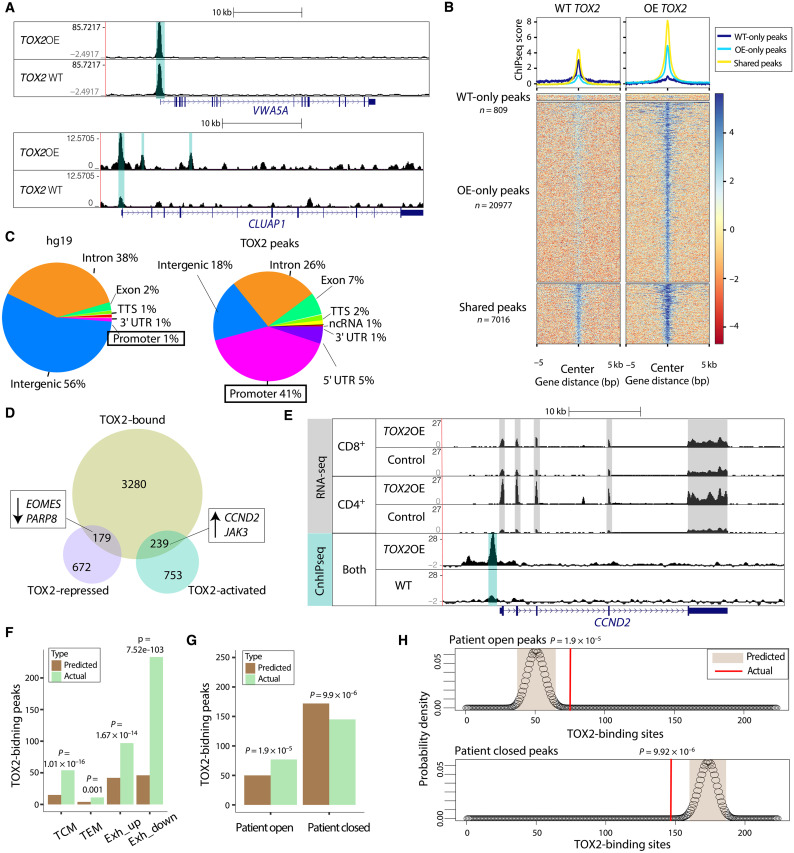
TOX2 binds to and activates central memory genes. (**A**) Chromatin immunoprecipitation with sequencing (ChIPseq) browser tracks for wild-type (WT) TOX2 (*n* = 1) and overexpressed TOX2 (*n* = 1) binding at the VWA5A and CLUAP1 loci, called peaks shaded in teal. Tracks visualized through http://genome.ucsc.edu. (**B**) Metaplot and heatmap of WT TOX2 (*n* = 1) and TOX2OE (*n* = 1) binding scores over the total number of peaks called for each sample alone and for their shared peaks. (**C**) Pie charts showing the makeup of the whole genome and the WT TOX2-bound sites, annotated by genomic region type. (**D**) Venn diagram showing the genes bound by TOX2 (*n* = 4238) and genes up- and down-regulated by TOX2 (*n* = 1843), with some memory- and exhaustion-related genes highlighted. (**E**) ChIPseq and RNA-seq browser tracks for control and overexpressed TOX2 at the *CCND2* locus, with MACS2-called ChIPseq peaks shaded in teal and exons shaded in gray. Tracks were visualized through http://genome.ucsc.edu. (**F**) Barplot of predicted number of TOX2 binding peaks versus actual number of TOX2 binding peaks for four gene sets of interest. Predictions calculated by multiplying the percentage of total promoters bound by TOX2 (4238 of 51794 = 8.1%) by the number of genes in each gene set (187, 49, 520, and 568, respectively), hypergeometric test *P* = 1.01 × 10^−16^, 0.001, 1.67 × 10^−14^, and 7.52 × 10^−103^. (**G**) Barplot of predicted number of TOX2 binding peaks versus actual number of TOX2 binding peaks for the open or closed assay for transposase accessible-chromatin using sequencing (ATACseq) peaks from the patient described in Fig. 1. Predictions calculated by multiplying the percentage of total changing ATAC peaks bound by TOX2 (222 of 32518 = 0.68%) by the number of genes in each gene set (7286 open peaks and 25,233 closed peaks), hypergeometric test, *P* = 1.9 × 10^−5^ and 9.9 × 10^−6^. (**H**) Probability density functions for hypergeometric tests shown in (G), comparing number of predicted TOX2 binding sites (tan) to actual number of TOX2-binding sites (red lines) for the peaks opened or closed in the patient.

Although TOX2 is classified as a transcription factor, little is known of how it functions at the level of chromatin. We investigated whether the location of TOX2 binding sites correlated with target genes regulated by TOX2. We performed chromatin immunoprecipitation with sequencing (ChIPseq) to determine TOX2 binding in the genome of human CAR T cells using T cells from two donors; in one set, we mapped endogenous levels of TOX2, and in the second set, we mapped overexpressed TOX2. Example browser tracks for both are shown at *VWA5A* and *CLUAP1* genes (Fig. 6A), which are both up-regulated in T_CM_ cells (*24*). In both ChIPseq datasets, numerous discrete binding peaks were identified using MACS2 (heatmaps and metaplots shown in Fig. 6B) (*28*). Over 90% of the 7755 peaks in the *TOX2* endogenous condition were replicated in the *TOX2* overexpression condition (fig. S4A), and *TOX2* overexpression led to an additional 20,364 peaks (fig. S4A). Further analyses were based on the consensus 7016 peaks in both samples (Fig. 6B, bottom). Compared to the entire genome (Fig. 6C, left), TOX2-bound peaks were highly enriched at promoter-TSS (transcription start site) regions (Fig. 6C, right). Furthermore, there was strong correlation between peak amplitude score and distance to TSS in that the strongest TOX2 peaks were closest to TSSs (fig. S4B), supporting an activation role for TOX2 bound at TSSs. Overall, the proximity of TOX2 binding to promoters strongly implicate TOX2 as a transcription factor.

We found that numerous genes regulated by TOX2 were also bound by TOX2. There were 1843 genes changed by either the *TOX2* knockdown, *TOX2* overexpression, or both; a total of 418 of these genes were bound by TOX2 within 5 kb of their promoters (Fig. 6D), including *ATP1A1*, *BAG2*, and *NOC3L*, all of which are up-regulated in T_CM_ over T_EM_ cells (*24*). Of the TOX2 peaks near promoters, 239 genes were activated by TOX2; some of these were also repressed by TOX, such as *CCND2* and *JAK3* (Fig. 6E), indicating that the functions of TOX2 that distinguish it from TOX involve specific genes bound by TOX2.

TOX2 also bound to 179 genes that it repressed, including *EOMES* and *PARP8* (Fig. 6D), which are expressed most highly in T_EM_ compared to other memory subsets, as well as up-regulated in T cell exhaustion (*24*). Thus, TOX2 may function as an activator at some genes and as a repressor at others. To delve deeper into the activity of TOX2 as an activator versus repressor, we analyzed all genes with TOX2 peaks within 1 kb of their promoters and compared the mean log_2_-fold change of these genes in the *TOX2* knockdown condition relative to the *TOX2* overexpression condition. *TOX2* overexpression led to a larger fold change, and these genes showed a trend toward activation by TOX2 (fig. S4C). This indicates that proximity of TOX2 binding to a gene promoter may positively correlate with TOX2-mediated activation of that gene.

To narrow our focus to genes of interest, we examined TOX2 binding at memory and exhaustion-related targets. TOX2 bound within 5 kb of 4238 distinct promoters, which is approximately 8.1% of total annotated promoters. We separated these 8.1% of promoters into four gene sets (T_CM_, T_EM_, exhaustion-up, and exhaustion-down) and then compared the predicted number to the actual number of TOX2-bound peaks. We found that although all four gene sets showed more TOX2 peaks than expected, the significance of the increase varied (Fig. 6F). Notably, T_CM_ genes and exhaustion-down genes showed much larger increases than T_EM_ genes and exhaustion-up genes (Fig. 6F), consistent with our earlier finding that a TOX2 peak near a promoter is more likely to lead to an activated gene (fig. S4C). Thus, the major targets of TOX2 activation are T_CM_ genes and genes that decrease in T_EX_.

Although it appears that TOX2 is more likely to bind to T_CM_ genes than exhaustion-up genes (Fig. 6F), this analysis was based on consensus peaks observed in both the endogenous TOX2 ChIPseq and the overexpressed TOX2 ChIPseq. Considering the acquisition of exhaustion gene expression that we observed with *TOX2* overexpression (Fig. 3G), we investigated whether overexpressed TOX2 bound more often to exhaustion-related genes than endogenous TOX2. For endogenous TOX2, the number of TOX2 peaks at exhaustion-up genes is barely higher than predicted by chance (fig. S4D), whereas for overexpressed TOX2, it is significantly higher (fig. S4E). This increase in binding to exhaustion genes may explain our findings that overexpressed TOX2 induces a T_PEX_ signature.

We compared our consensus TOX2 binding data to the ATACseq data from the patient described in Fig. 1, who responded completely to CAR T therapy. We found that TOX2 is much more likely than predicted by chance to bind to regions open in the patient (Fig. 6, G and H). In contrast, the number of TOX2 peaks at closed regions was less than predicted by chance (Fig. 6, G and H). Together, these data show that TOX2 binding is enriched at regions of open chromatin in a patient who responded to CAR T therapy and suggest that TOX2 may play a role in increasing T_CM_ differentiation during the antitumor response.

## DISCUSSION

Our results show that a remarkable patient response to CAR T cell therapy (*7*) correlates with an increase in chromatin accessibility at both *TOX* and *TOX2* (Fig. 1D). This is unexpected, given that, in preclinical mouse studies, they are identified as redundant regulators of T cell exhaustion (*9*). Our in vitro stress test model, incorporating human CAR T cells, showed that TOX2 contributes to the formation and proliferation of T_CM_ in response to antigen (Fig. 2, C and E), which predict robust CAR T cell function. However, we find that, while high levels of TOX2 expression are sufficient to increase central memory CAR T cell differentiation (Fig. 3B), they do not improve proliferation (Fig. 3D), possibly because of the up-regulation of exhaustion precursor pathways (T_PEX_; Fig. 3, F and G). In addition, we have found that TOX2 binds chromatin at many of the genes it regulates, including key central memory and exhaustion-related genes (Fig. 6D). In clear contrast, our results show that TOX works differently, wherein loss of TOX leads to increases in T_CM_ and their accompanying transcriptomic programs (Fig. 5, B, D, and E).

Hence, we identify a role for TOX2 distinct from TOX as a regulator of memory differentiation in human CAR T cells. First, TOX2 is necessary for maximal differentiation of central memory cells (Fig. 2B), while loss of TOX leads to an increase in central memory cells (Fig. 5B), suggesting that TOX2 promotes central memory cell differentiation, whereas TOX promotes exhausted T cells. Thus, while TOX and TOX2 are both HMG box transcription factors and are highly similar in protein sequence (*29*), they are not functionally redundant in human T cells; their highly differential effects on central memory cells have implications for the design of CAR T therapies. Notably, our findings also indicate that despite their high sequence similarity, TOX and TOX2 drive expression of different genes, which likely explain their distinct functions. The mechanisms of DNA binding and protein interactions underlying distinct function of TOX and TOX2 remain to be investigated.

Unlike previous studies of exhausted T cells that focused primarily on CD8^+^ T cells, we investigated CD4^+^ and CD8^+^ T cells. We found that the effects of *TOX2* knockdown and overexpression were greater in CD4^+^ compared to CD8^+^ (Fig. 2, H to J, and fig. S1, B and C). Thus, together with our recent findings that CD4^+^ cells persist throughout prolonged patient remission after CAR T therapy (*2*), these results highlight the importance of analyzing CD4^+^ cells in the context of CAR T therapy.

There is strong consensus from various studies that TOX is required for the formation of exhausted T cells (*8*, *9*, *30*, *31*); however, mechanisms by which TOX regulates memory-related gene expression are not fully understood. Strangely, in a mouse model of chronic viral infection, *Tox*^−/−^ CD8^+^ T cells show down-regulation of memory-related genes, such as *Sell*, *Ccr7*, *Tcf7*, and *Lef1* (*8*). This suggests that some function of TOX may be required for central memory cells. However, our findings in human CAR T cells show *TOX* knockdown up-regulating *SELL*, *CCR7*, *TCF7*, and *LEF1* (fig. S3E), which is consistent with our findings that loss of TOX increases central memory cells (Fig. 5B). Thus, TOX may function beyond exhaustion regulation, and these functions may be context dependent. Most notably, past studies in mouse show TOX loss down-regulating *Tox2* (*8*), whereas we detect loss of TOX up-regulating *TOX2* (Fig. 5F), further emphasizing TOX2 function in human T_CM_.

In conclusion, our research highlights the importance of TOX2 as a key regulator of T_CM_ differentiation in human CAR T therapy. Our results show that TOX2 promotes formation of T_CM_, which are key predictors of robust CAR T cell function. However, TOX2 overexpression does not result in improved CAR T function, likely because of the emergence of exhaustion precursor cells. Our findings thus indicate that there is an optimal level of TOX2 expression that might be achievable via manipulating transgene delivery to harness the benefits of TOX2 while avoiding the activation of exhaustion pathways. These results provide insights into how TOX2 can be used to improve the effectiveness of CAR T therapy and pave the way for further research in this area.

## MATERIALS AND METHODS

### ATACseq data analysis

Raw data were downloaded from the Gene Expression Omnibus (accession number GSE112494). Data were trimmed of adapter contamination using Cutadapt (*32*). They were aligned to GRC37/hg19 using bowtie2 (*33*). After removing mitochondrial reads, polymerase chain reaction (PCR) duplicates were removed with Picard. Peaks were called with MACS2 (*28*). Browser tracks were made with bedtools (*34*) and visualized using the UCSC Genome Browser (*35*). Differential peaks were assessed using the Bioconductor package DiffBind (*36*, *37*). Peaks were annotated using annotatePeaks.pl from Homer (*38*).

### Plasmids

We used the CD19BBζ CAR plasmid, and lentiviral packaging plasmids were provided by the Center for Cellular Immunotherapies at the Perelman School of Medicine. The sh*TOX2* and sh*TOX* plasmids were designed and made in the Product Development Laboratory at the Center for Cellular Immunotherapies. The pMSCV-IRES-mCherry plasmid was also provided by the Center for Cellular Immunotherapies, and the TOX2 sequence was cloned into it. The retroviral packaging plasmids were provided by C. June. The *TOX2* sequence was generated by PCR amplification of cDNA that was reverse-transcribed from mRNA isolated from human 293T cells. The pLPC-FLAG plasmid, described previously (*39*), was used to clone the same *TOX2* cDNA sequence.

### Cell lines and culture conditions

293T, IMR90, and SupT1 cells were purchased from the American Type Culture Collection. K562-CD19 and K562-mesothelin cells were provided by J. Fraietta. 293T cells were cultured in Dulbecco’s modified Eagle’s medium (DMEM; Gibco), supplemented with 10% fetal bovine serum (FBS; Gibco) and 5% penicillin-streptomycin (Gibco) in a humidified incubator at 37°C. SupT1 and K562 cells were cultured in RPMI 1640 (Gibco) supplemented with 10% FBS and 5% penicillin-streptomycin, incubated as above. IMR90 cells were cultured in DMEM supplemented with 10% FBS and 5% penicillin-streptomycin, cultured under physiological oxygen (3%).

### Lentiviral vector packaging

Log-phase 293T cells were seeded at 1 × 10^7^ cells per 175-cm^2^ flask. After 24 hours, 15 μg of vector plasmid, 18 μg of pRSV.REV (encoding Rev), 18 μg of pMDLg/p.RRE (encoding Gag/Pol), and 7 μg of pVSV-G (encoding vesicular stomatitis virus glycoprotein) were mixed with 90 μl of Lipofectamine 2000 (Invitrogen) and 1.5 ml of Opti-MEM I Reduced Serum Medium (Invitrogen) and incubated at room temperature for 30 min and then added to the 293T cells. After 24 hours, supernatant was harvested, passed through a 0.45-μm filter, and concentrated by overnight centrifugation at 8500 rpm. Fresh medium was added to the 293T cells, and supernatant was harvested, filtered, and concentrated again after another 24 hours. The concentration of lentivirus was determined by infecting SupT1 cells at concentrations of viral supernatant ranging from 1:3 to 1:6561 and then staining for the vector, identifying the percent positively stained by flow cytometry, and calculating the titer at each concentration using the formula: Titer (TU/ml) = (% positive/100) × 2 × 10^4^ × 20 × dilution. The titer from the highest dilution with less than 20% positive cells was used to calculate multiplicity of infection (MOI).

### Generation of human CAR T cells

Human peripheral blood mononuclear cells (PBMCs) from healthy adult donors were purchased from the Abramson Cancer Center’s Human Immunology Core. CD3^+^ T cells were isolated from PBMCs using the EasySep Human T Cell Isolation Kit (STEMCELL Technologies), following the manufacturer’s instructions. Cells were placed in Opt5 media [5% heat-inactivated GemCell Human Serum AB (Gemini Bio) and 2 mM GlutaMAX (Gibco) in CTS OpTmizer T Cell Expansion SFM (Gibco)] at a concentration of 1 × 10^6^ cells/ml and activated with Dynabeads Mouse T-Activator CD3/CD28 (Gibco) at a ratio of 3:1 beads to cells and human recombinant IL-2 (100 IU/ml; PeproTech Inc.). One day after activation, cells were infected with CAR19 containing lentivirus at an MOI of 2. On days 5, 7, 9, and 12, cells were counted using a Countess automated cell counter; then, Opt5 with IL-2 was added to bring the cell concentration down to 5 × 10^5^ cells/ml. Cells were harvested on day 14.

### shRNA knockdown

CAR T cells were manufactured as above. One day after activation, cells were infected with shRNA-containing lentivirus at an MOI of 3 and then expanded as above.

### Retroviral overexpression in CAR T cells

Log-phase 293T cells were seeded at 8 × 10^6^ cells per 15-cm plate. After 24 hours, 40 μg of pMSCV vector plasmid, 20 μg of plasmid encoding retrovirus-specific Gag/Pol, and 20 μg of plasmid encoding RD114 envelope protein were mixed with 90 μl of Lipofectamine 2000 and 3 ml of Opti-MEM and incubated at room temperature for 30 min and then added to the 293T cells. After 2 days, viral supernatant was harvested and passed through a 0.45-μm filter, and fresh medium was added. Twenty-four hours later, the final viral supernatant was harvested and filtered. Nontissue-culture treated six-well plates were filled with RetroNectin (20 μg/ml; Takara Bio) in phosphate-buffered saline (PBS) and then left at 4°C overnight. The plates were blocked with 2% bovine serum albumin in PBS for 30 min and then washed with PBS. Viral supernatant was added, and plates were centrifuged for 2 hours at 2000*g*. CAR T cells were generated as above. Four days after activation, they were added to the prepared viral plates. After two more days, the T cells were placed in fresh viral plates for a second round of infection and then expanded until day 14 as above.

### Flow cytometry

All flow cytometry experiments were performed on an LSRFortessa (BD Biosciences). For each panel, 1 × 10^6^ cells were stained with LIVE/DEAD Fixable Aqua Dead Cell Stain Kit (Thermo Fisher Scientific) in 100 μl of PBS for 15 min at room temperature in the dark. Cells were washed with 200 μl of fluorescence-activated cell sorting (FACS) buffer (0.5% Human Serum AB and 1 mM EDTA in PBS). Cells were stained with antibodies in 100 μl of FACS buffer for 20 min at room temperature in the dark, washed with 200 μl of FACS buffer, resuspended, and then analyzed. The CD19-CAR was stained with biotin-conjugated anti-mouse F(ab′)_2_ antibody (Jackson ImmunoResearch), washed, and then labeled with APC-streptavidin (BioLegend). Other marks were assessed using the following antibodies: CD27-phycoerythrin (PE)/cyanine7 (clone O323), PD-1–Brilliant Violet (BV)421 (clone EH12.2H7), CD45RO-BV570 (clone UCHL1), CD8a-BV650 (clone RPA-T8), and CD4-BV785 (clone OKT4) (from BioLegend) and CCR7–fluorescein isothiocyanate (clone 150503) and CD3-allophycocyanin(APC)-H7 (clone SK7) (from BD Pharmingen).

### Fluorescence-activated cell sorting

All sorting was performed on a FACSAria II (BD Biosciences). Up to 1 × 10^8^ frozen CAR T cells were thawed and washed in sterile FACS buffer, then resuspended in 1 ml of FACS and stained with antibodies, and then sorted. For the antigen restimulation experiments, the staining was carried out in sterile conditions: stained with LIVE/DEAD Fixable Aqua for 15 min in the dark, washed in 1 ml of FACS buffer, stained with biotin-conjugated F(ab′)_2_ for 20 min, washed, stained with APC-H7-CD3 and APC-streptavidin, washed, resuspended in 1 ml of FACS buffer, and sorted. For the RNA-seq experiments, the staining was carried out as above, with the addition of CD4-BV785 and CD8a-BV650 in the final antibody staining step. Sorting was performed with a 100-μm nozzle, and cells were collected into a solution of 50% Human Serum AB and 50% OpTmizer.

### Antigen restimulation

Either 5 × 10^5^ or 1 × 10^6^ sorted CAR T cells were cocultured in cytokine-free Opt5 media at a 1:1 ratio with gamma-irradiated K562-CD19 cells or gamma-irradiated K562-mesothelin cells, with a total cell concentration of 1 × 10^6^ per ml. After 7 days, cells were counted with a Countess automated cell counter, gating for cells between 3 and 15 μm in diameter. Then, 5 × 10^5^ cells were reseeded in 1 ml of fresh Opt5 with 5 × 10^5^ target cells. This process was repeated on days 12, 17, 22, and 27. At each time point, cell count, size, and viability were recorded.

### Retroviral overexpression in IMR90 cells

Phoenix retroviral packaging cells were seeded at 1.6 × 10^6^ cells per 6-cm plate. After 24 hours, 2.5 μg of vector plasmid was mixed with 500 μl of Opti-MEM and 7 μl of Lipofectamine 2000, incubated for 20 min, and then added to the cells. After 12 hours, viral supernatant was harvested, and fresh medium was added. This was repeated after 24 hours, and log-phase IMR90 cells were seeded at 2 × 10^5^ cells per well of a six-well plate. After 18 hours, the final viral supernatant was harvested, and all harvests were pooled and passed through a 0.45-μm filter. Four milliliters of virus plus 1:1000 polybrene (Millipore) were added to each well of IMR90 cells for 12 hours, followed by 12 hours in fresh medium. This cycle of infection was repeated two more times. Four days after the first infection, cells were treated with puromycin (500 ng/μl) and then grown until noninfected control cells were all dead.

### RNA sequencing

Cells were frozen at −80°C in TRIzol (Ambion); then, RNA was extracted using the RNA Clean and Concentrator kit (Zymo Research). mRNA was isolated using the NEBNext Poly(A) mRNA Magnetic Isolation Module (NEB), and RNA-seq libraries were made using NEBNext Ultra II Directional Library Prep Kit for Illumina (NEB). Library sizes were determined on a Bioanalyzer, and concentrations were calculated with the NEBNext Lib Quant Kit (NEB). Libraries were pooled and sequenced on an Illumina NextSeq 550 using paired-end sequencing of 42 bases per read.

### RNA-seq data analysis

RNA-seq data were aligned to the reference human genome assembly, GRC37/hg19, using STAR (*40*). Reads for each gene with RefSeq annotations were counted using htseq-counts or featureCounts (*41*, *42*). Counts were normalized, and significant differences were calculated with DESeq2 (*43*).

### ChIPseq

A total of 2.5 × 10^7^ T cells were cross-linked for 15 min at room temperature with 1% formaldehyde in PBS. Cross-linking was quenched with 125 mM glycine. Nuclei were isolated by lysing cells for 10 min at 4°C in 50 mM Hepes, 140 mM NaCl, 1 mM EDTA, 10% glycerol, 0.5% NP-40, and 0.25% Triton X-100 and then pelleting and lysing again in 10 mM tris-HCl (pH 8.0), 200 mM NaCl, 1 mM EDTA, and 0.5 mM EGTA. After pelleting again, nuclei were lysed in 10 mM tris-HCl (pH 8.0), 100 mM NaCl, 1 mM EDTA, 0.5 mM EGTA, 0.1% Na-deoxycholate, and 0.5% *N*-lauroylsarcosine, and chromatin was sheared using a Covaris S220 sonicator. After decross-linking a small sample at 65°C overnight, DNA was extracted using the QIAquick Gel Extraction Kit (QIAGEN) and quantified on a Qubit Fluorometer. For each immunoprecipitation, 30 μl of Dynabeads Protein G were incubated rotating overnight at 4°C with 5 μg of TOX2 antibody (Invitrogen, PA5-62084). After washing, the beads were combined with a quantity of lysate that contained 5 μg of DNA and rotated overnight at 4°C. After five washes with radioimmunoprecipitation assay buffer, DNA was eluted from the beads by incubation at 65°C for 30 min into 50 mM tris-HCl (pH 8.0), 10 mM EDTA, and 1% SDS. After reversing the cross-linking, DNA was purified by phenol-chloroform extraction followed by ethanol precipitation. ChIPseq libraries were made from 3 μg of the immunoprecipitated DNA and input lysis controls with the NEBNext Ultra II DNA Library Prep Kit (NEB). Library sizes were determined on a Bioanalyzer, and concentrations were calculated with the NEBNext Lib Quant Kit (NEB). Libraries were pooled and sequenced on an Illumina NextSeq 550 using paired-end sequencing of 42 bases per read.

### ChIPseq data analysis

ChIPseq data were trimmed of adapter contamination using Cutadapt (*32*). They were aligned to GRC37/hg19 using bowtie2 (*33*). After removing mitochondrial reads, PCR duplicates were removed with Picard. Peaks were called with MACS2 (*28*) using the input files as background. Input-subtracted bigWig files were made with bedtools genomeCoverageBed and bedGraphtoBigWig (*34*). Tracks were visualized using the UCSC Genome Browser (*35*). Metaplots and heatmaps were produced with deeptools (*44*). Peaks were annotated using annotatePeaks.pl (including the -genomeOntology option for comparisons to the entire genome) from Homer (*38*).

### Statistical analysis

For two-sample comparisons of means, a paired Student’s *t* test was used, and significance was established by a *P* value ≤0.05. For GSEA, significance was established by a false discovery rate (FDR *q* value) ≤0.25, per the developer’s instructions. To assess the enrichment of ChIPseq binding at various peak sets (both ChIPseq and ATACseq peaks), the R package phyper was used to perform hypergeometric tests, and significance was established by a *P* value ≤0.05.

## Notes

View/request a protocol for this paper from *Bio-protocol*.
