# Supplementary material for: TOX2 coordinates with TET2 to positively regulate central memory differentiation in human CAR T cells
Source: Sci Adv. 2023 Jul 19;9(29):eadh2605. doi: 10.1126/sciadv.adh2605 (PMC10355826; doi:10.1126/sciadv.adh2605)
Supplement: Supplementary file 1 — Figs. S1 to S4 Tables S1 to S8, and S10 Legend for table S9 [file sciadv.adh2605_sm.pdf]

Science Advances
AAAS

Supplementary Materials for

TOX2 coordinates with TET2 to positively regulate central memory differentiation in human CAR T cells

Sierra M. Collins *et al.*

Corresponding author: Joseph A. Fraietta, jfrai@upenn.edu; Shelley L. Berger, bergers@pennmedicine.upenn.edu

Sci. Adv. **9**, eadh2605 (2023)
DOI: 10.1126/sciadv.adh2605

The PDF file includes:

Figs. S1 to S4
Tables S1 to S8, and S10
Legend for table S9

Other Supplementary Material for this manuscript includes the following:

Table S9

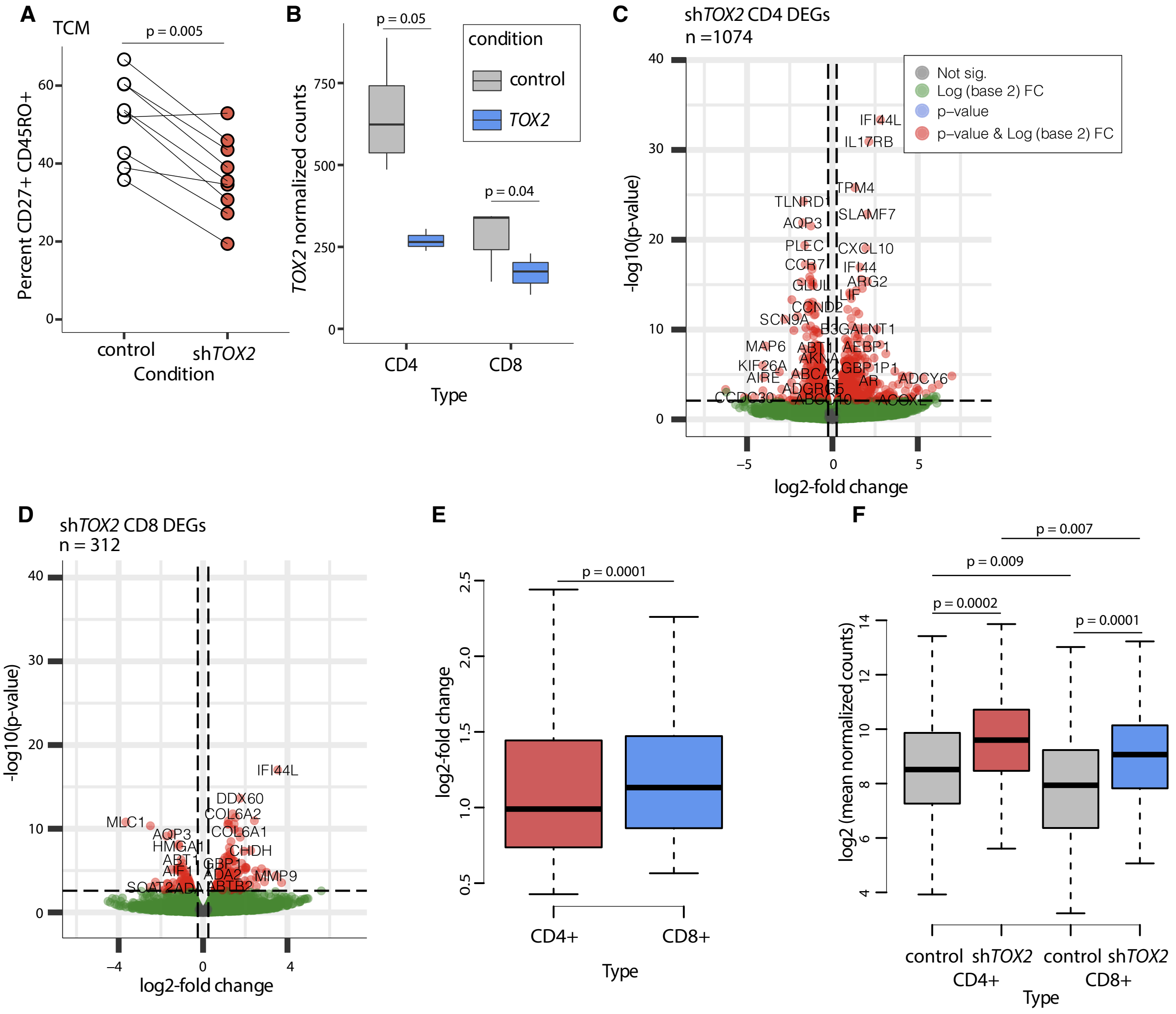

Fig. S1. Loss of TOX2 affects CD4^{+} cells more than CD8^{+}.
(A) Percentage of CD45RO+ CD27+ CAR19+ T cells in 7 human donors over 4 separate experiments, 12 samples total. (B) Normalized RNAseq counts for TOX2 in sorted CD4+ and CD8+ CAR+ human T cells, with or without the shRNA targeting TOX2. (C) Volcano plot showing log2 fold-change vs. –log10(p.value) of all genes in CD4+ CAR+ T cells, with padj < 0.1 and FC > 0.25 (calculated by DESeq [43]) considered significant. (D) Volcano plot showing log2 fold-change vs. -log10(p.value) of all genes in CD8+ CAR+ T cells, with padj < 0.1 and FC > 0.25 (calculated by DESeq [43]) considered significant. (E) Average log2 fold-change in the shTOX2 vs. control for the 137 genes upregulated in both CD4+ and CD8+ T cells, shown as boxplots in CD4+ and CD8+ T cells. (F) Average normalized RNAseq counts for the 137 genes upregulated in both CD4+ and CD8+ T cells, shown as boxplots of log2 (mean mRNA count) in each condition.

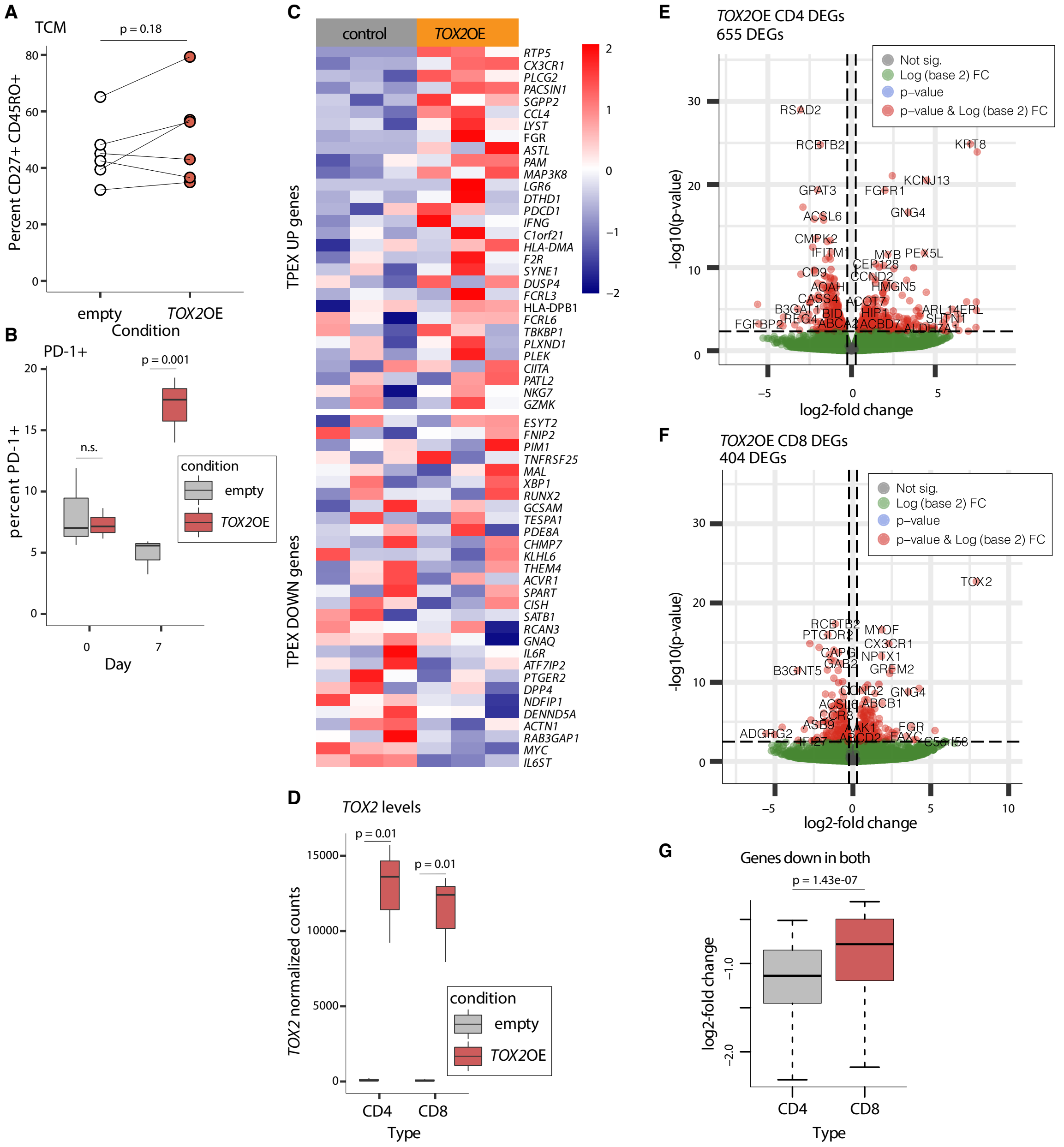

Fig. S2. TOX2 overexpression promotes genes upregulated in TPEX.

(A) Percentage of CD45RO+ CD27+ CAR19+ T cells in 5 human donors over 4 separate experiments, 7 samples total. (B) Percentage of PD-1+ cells after 0 or 7 days of co-culture with CD19-presenting K562 cells, comparing control vs. TOX2OE. (C) Heatmap of z-scores of RNAseq normalized counts from empty vs. TOX2OE T cells for genes up- (top) and downregulated (bottom) in TPEX. (D) Normalized RNAseq counts for TOX2 in sorted CD4+ and CD8+ CAR+ human T cells, with or without the TOX2 overexpression construct. (E) Volcano plot showing log2 fold-change vs. –log10(p.value) of all genes in CD4+ CAR+ T cells, with padj < 0.1 and FC > 0.25 (calculated by DESeq [43]) considered significant. (F) Volcano plot showing log2 fold-change vs. –log10(p.value) of all genes in CD8+ CAR+ T cells, with padj < 0.1 and FC > 0.25 (calculated by DESeq [43]) considered significant. (G) Boxplots of log2-fold change for genes downregulated in both CD4+ and CD8+ T cells with TOX2 OE.

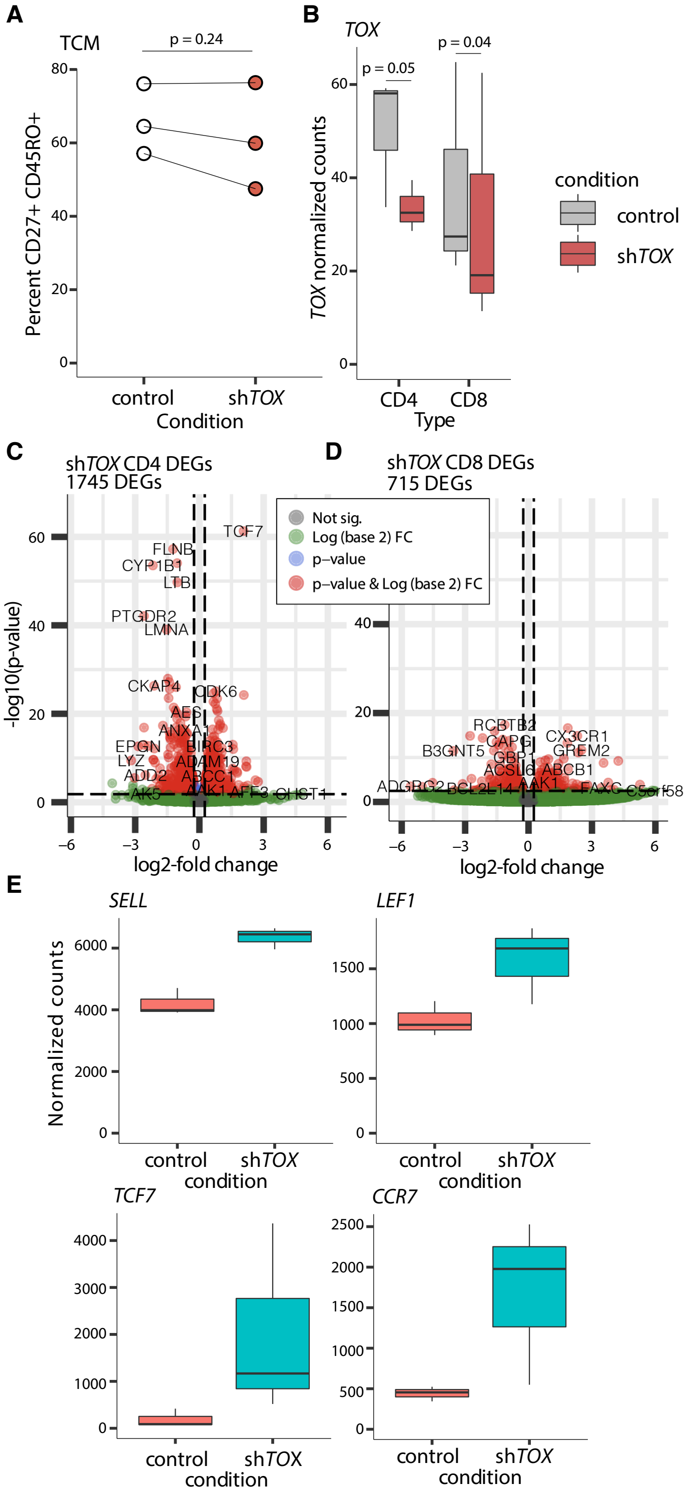

Fig. S3. Loss of TOX increases memory gene expression.

(A) Percentage of CD45RO+ CD27+ CAR19+ T cells in 3 human donors, paired Student's t-test, P=0.24. (B) Normalized RNAseq counts for TOX in CD4+ and CD8+ CAR19+ T cells, control vs. shTOX, 3 human donors, paired Student's t-test, P=0.05 and 0.04. (C) Volcano plot showing log2 fold-change vs. –log10(p.value) of all genes in CD4+ CAR+ T cells, with padj < 0.1 and FC > 0.25 (calculated by DESeq [43]) considered significant. (D) Volcano plot showing log2 fold-change vs. –log10(p.value) of all genes in CD8+ CAR+ T cells, with padj < 0.1 and FC > 0.25 (calculated by DESeq [43]) considered significant. (E) Boxplots showing normalized RNAseq counts of various memory genes in control vs. shTOX, 3 human donors, paired Student's t-test, P=0.02, 0.04, 0.11, and 0.07.

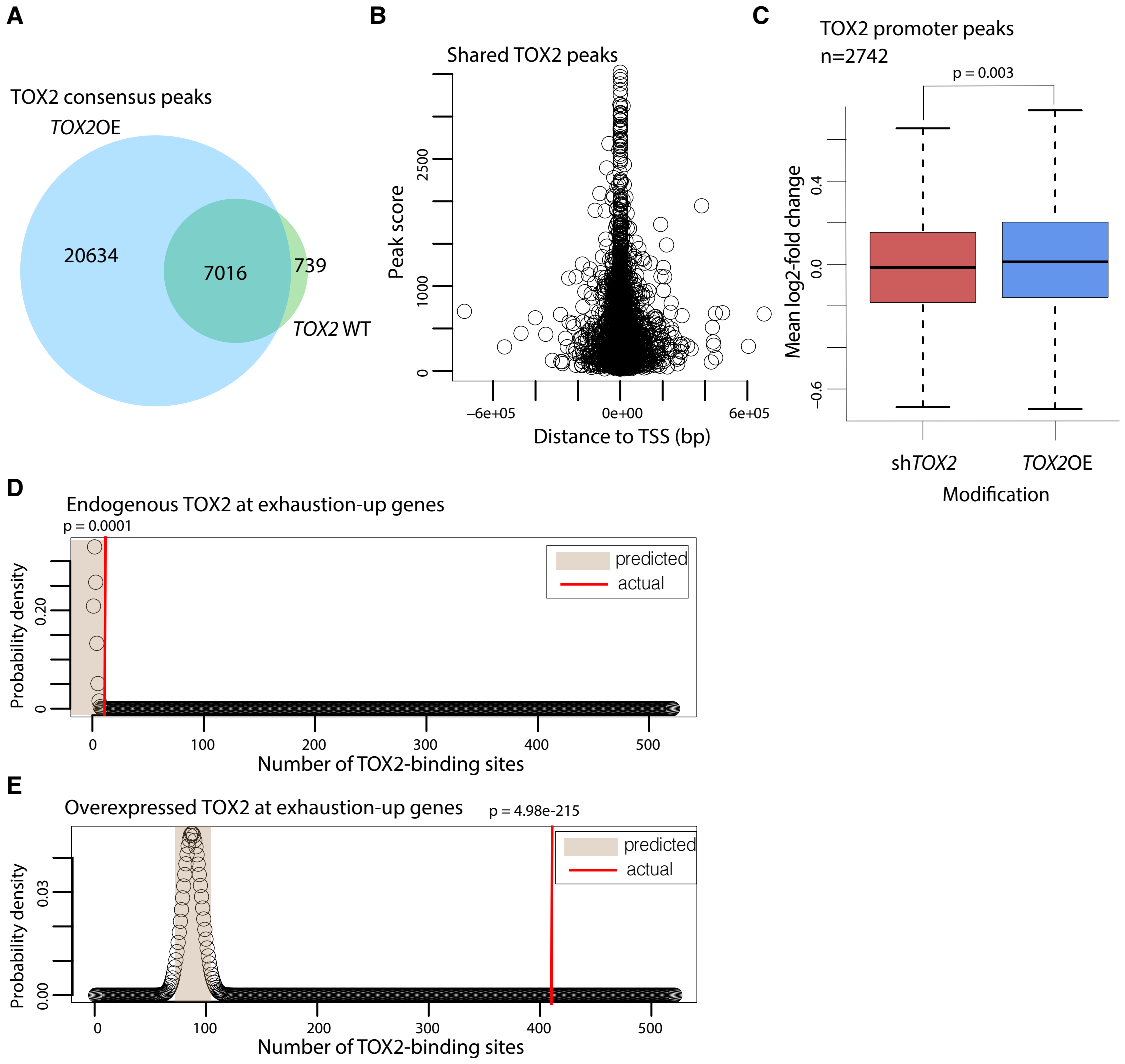

Fig. S4. TOX2 overexpression results in more binding to exhaustion-related genes.

(A) Venn diagram showing the overlap (n=7016) between peaks called in the WT TOX2 ChIPseq (n=7755) and the TOX2 OE ChIPseq (n=27383). (B) Peak distance to TSS (base pairs) vs. ChIPSeq peak score for peaks called in both the WT and TOX2OE ChIPseq data. (C) Boxplots showing the log2fc in the CD4+ T cells TOX2KD and TOX2OE conditions for all genes with TOX2 peaks within 1kb of the TSS. Unpaired Student's t-test, P=0.003. (D) Probability density functions for hypergeometric test comparing the number of predicted endogenous TOX2 binding sites (tan) to actual number of endogenous TOX2-binding sites (red lines) at genes upregulated in exhaustion, P=0.0001. (E) Probability density functions for hypergeometric test comparing the number of predicted OE TOX2 binding sites (tan) to actual number of OE TOX2-binding sites (red lines) at genes upregulated in exhaustion, P=4.98e-215.

Table S1

Percent	Population	Donor	Condition
41.1	TCM	365	control
36	TCM	436	control
35.1	TCM	365	control
11.4	TCM	502	control
21.7	TCM	528	control
18.7	TCM	317	control
29.5	TCM	365	control
23.5	TCM	502	control
16.2	TCM	566	control
22.9	TCM	502	control
42.6	TCM	528	control
59.8	TCM	569	control
22.9	TCM	365	TOX2KD
19.8	TCM	436	TOX2KD
14.1	TCM	365	TOX2KD
9.2	TCM	502	TOX2KD
15	TCM	528	TOX2KD
8.92	TCM	317	TOX2KD
9.7	TCM	365	TOX2KD
6.54	TCM	502	TOX2KD
10.3	TCM	566	TOX2KD
16.4	TCM	502	TOX2KD
21.9	TCM	528	TOX2KD
34.4	TCM	569	TOX2KD
54	TEM	365	control
58	TEM	436	control
50.5	TEM	365	control
62.6	TEM	502	control
62.9	TEM	528	control
76.5	TEM	317	control
70.2	TEM	365	control
72.3	TEM	502	control
80.2	TEM	566	control
62.9	TEM	502	control
49.5	TEM	528	control
33.7	TEM	569	control
75.9	TEM	365	TOX2KD
78.8	TEM	436	TOX2KD
81.4	TEM	365	TOX2KD
79.5	TEM	502	TOX2KD
80.3	TEM	528	TOX2KD
90	TEM	317	TOX2KD
90	TEM	365	TOX2KD
90.3	TEM	502	TOX2KD
88.7	TEM	566	TOX2KD
75.1	TEM	502	TOX2KD
74.7	TEM	528	TOX2KD
63.1	TEM	569	TOX2KD

Percent of TCM and TEM cells for control (*N*=12) or shTOX2 (N=12) conditions. Each TCM line represents the CCR7+ CD45RO+ population from a separate experiment. Each TEM line represents the CCR7- CD45RO+ population from those same experiments.

Table S2

Percent of CD27+ CD45RO+ T cells in various conditions. Each line is a separate experiment.

Percent	Donor	Condition
35.8	365	control
19.4	365	TOX2KD
53.2	436	control
30.7	436	TOX2KD
60.3	365	control
43.5	365	TOX2KD
38.9	502	control
34.6	502	TOX2KD
51.9	528	control
52.9	528	TOX2KD
53.7	317	control
35.5	317	TOX2KD
42.7	365	control
27.2	365	TOX2KD
60.4	502	control
39	502	TOX2KD
66.7	566	control
45.9	566	TOX2KD
39.4	317	control
65.1	365	control
45.1	436	control
48.2	528	control
32.2	561	control
42.5	578	control
57	317	TOX2OE
79.3	365	TOX2OE
43	436	TOX2OE
56.3	528	TOX2OE
34.9	561	TOX2OE
36.6	578	TOX2OE
76.1	365	control
64.5	528	control
57.1	578	control
76.4	365	TOXKD
59.9	528	TOXKD
47.5	578	TOXKD

Table S3

Condition		control			shTOX2		
**Day**	**Antigen**	**ND317**	**ND365**	**ND436**	**ND317**	**ND365**	**ND436**
**0**	**CD19**	0	0	0	0	0	0
**7**		2.26303441	1.58111018	0.83592407	1.76553475	1.50944249	1.12267272
**12**		3.28622403	2.42137143	3.04277564	2.50250034	3.01728057	2.14086044
**17**		4.59656415	4.69589763	3.28235677	4.25096157	2.98404388	2.39597705
**22**		4.85959856	6.38286332	5.01416066	3.41446031	4.62009732	2.40235046
**27**		2.53767046	5.84110532	3.35986272	2.066537	3.97360494	2.67448397
**0**	**mesothelin**	0	0	0	0	0	0
**7**		0.37851162	0.59645814	0.73811929	0.01435529	0.31892416	0.05547334
**12**		0	-0.2331081	0.12252695	0	-0.6690413	-0.4499425
**17**		1.37851162	-4.0558055	-0.1270503	0.37851162	-0.949712	0.31915501
**22**		-0.577767	-1.9775541	0.69383778	0.5849625	-0.294702	1.07618865

Population doublings in control (*N*=3) or shTOX2-treated (*N*=3) normal donor (ND) CAR T cells stimulated with either CD19 or mesothelin, measured over 22 days.

Table S4

Condition	Cell type	NAME	SIZE	NES	NOM p-val	FDR q-val
TOX2KD	CD4	EXHAUSTED_UP	409	-1.5886586	0	0
control	CD4	EXHAUSTED_DOWN	540	1.7252814	0	0
TOX2KD	CD8	EXHAUSTED_UP	409	1.5152785	0	0
control	CD8	EXHAUSTED_DOWN	540	-2.5764015	0	0
TOX2KD	CD8	GSE11057_EFF_MEM_VS_CENT_MEM_CD4_TCELL_DN	167	1.4661182	0.00531209	0.00531209
control	CD4	TN LOWEST	725	1.5869929	0	0.01812005
TOX2KD	CD8	TN HIGHEST	86	1.5568491	0.00742942	0.02229999
control	CD4	TCM/TCSM	11	1.6020699	0.02739726	0.02538793
TOX2KD	CD8	TCM LOWEST	25	1.6085002	0.01085271	0.02966666
TOX2KD	CD8	TN/TCM	31	1.7494391	0.00325203	0.02999048
control	CD4	TCSM/TEM	20	1.6202444	0.02056555	0.03109561
TOX2KD	CD4	TN HIGHEST	86	-1.5427724	0.00143472	0.03253571
control	CD4	TSCM/TN	51	1.5037545	0.02064897	0.03935734
TOX2KD	CD8	TEM HIGHEST	39	1.4071317	0.0609375	0.04493749
TOX2KD	CD8	TEM LOWEST	291	1.4357162	0.00241838	0.04677778
control	CD4	TEM/TCSM	39	1.4454876	0.03197675	0.04970678
control	CD4	TCM/TN	96	1.6263462	0	0.05645398
control	CD4	GSE11057_EFF_MEM_VS_CENT_MEM_CD4_TCELL_DN	167	-1.1989652	0.06048387	0.06048387
TOX2KD	CD4	TEM LOWEST	291	-1.4030806	0.00956938	0.0699107
TOX2KD	CD4	GSE11057_EFF_MEM_VS_CENT_MEM_CD4_TCELL_UP	175	1.2339215	0.09125964	0.09125964
TOX2KD	CD8	TEM/TN	117	1.4862514	0.00828729	0.15911418
TOX2KD	CD8	GSE11057_EFF_MEM_VS_CENT_MEM_CD4_TCELL_UP	175	1.168878	0.16433121	0.16433121
TOX2KD	CD8	GSE23321_CENTRAL_VS_EFFECTOR_MEMORY_CD8_TCELL_UP	169	1.1469857	0.19480519	0.19480519
TOX2KD	CD4	TCM LOWEST	25	-1.1494781	0.2782462	0.3004361
TOX2KD	CD4	TEM HIGHEST	39	-1.0470936	0.3828756	0.3634928
TOX2KD	CD8	TN/TCSM	15	1.2026601	0.21847247	0.404231
control	CD4	TCM/TN	199	0.97627777	0.544	0.52098876
TOX2KD	CD8	TN/TEM	61	1.0440596	0.36818182	0.5218692
TOX2KD	CD8	TCM/TN	96	0.972937	0.51324964	0.57607764
control	CD8	GSE23321_CENTRAL_VS_EFFECTOR_MEMORY_CD8_TCELL_DN	176	-0.9250029	0.6653226	0.6653226
TOX2KD	CD4	TEM/TN	117	-1.0533216	0.35612535	0.77040356
TOX2KD	CD4	TN/TCSM	15	-0.8869507	0.6084034	0.77160317
control	CD8	TCM/TCSM	11	-0.8095132	0.69248825	0.7791181
control	CD8	TSCM/TN	51	-0.9455421	0.55	0.8076353
TOX2KD	CD8	TCSM/TEM	20	0.7567478	0.80431175	0.8416844
TOX2KD	CD4	TN/TEM	61	-0.9298717	0.58084774	0.8660419
TOX2KD	CD8	TCM/TN	199	0.7375588	0.9714994	0.9475983
TOX2KD	CD8	TN LOWEST	725	0.8370135	0.9636163	0.9669645
TOX2KD	CD4	TN/TCM	31	-1.054445	0.37766832	1
control	CD8	TEM/TCSM	39	-1.037143	0.38502672	1

Gene set enrichment analysis (GSEA) results for RNAseq counts from control or shTOX2 samples (*N*=3 for each group), in both $CD4^+$ and $CD8^+$ T cells. Gene set size, normalized enrichment score (NES), nominal p-value, and false discovery rate (FDR) q-values are reported. FDR q-value < 0.25 was considered significant. Gene sets are referenced in main text.

Table S5

Condition	Population	Percent	Donor
empty	TCM	14.8	528
empty	TCM	9.93	561
empty	TCM	18.5	578
empty	TCM	39.7	317
empty	TCM	57.5	365
empty	TCM	23.4	317b
empty	TCM	22.7	365b
TOX2OE	TCM	18.4	528
TOX2OE	TCM	15.1	561
TOX2OE	TCM	28.4	578
TOX2OE	TCM	48.6	317
TOX2OE	TCM	70.3	365
TOX2OE	TCM	54.8	317b
TOX2OE	TCM	40.5	365b
empty	TEM	76.9	528
empty	TEM	83.7	561
empty	TEM	76.8	578
empty	TEM	24.6	317
empty	TEM	34.4	365
empty	TEM	76.3	317b
empty	TEM	77.2	365b
TOX2OE	TEM	75.6	528
TOX2OE	TEM	79.5	561
TOX2OE	TEM	69.6	578
TOX2OE	TEM	20.7	317
TOX2OE	TEM	22	365
TOX2OE	TEM	44.1	317b
TOX2OE	TEM	59.2	365b

Percent of TCM and TEM cells for control (N=7) or TOX2OE (N=7) conditions. Each TCM line represents the CCR7+ CD45RO+ population from a separate experiment. Each TEM line represents the CCR7- CD45RO+ population from those same experiments.

Table S6

Condition	control			TOX2OE		
Day	ND317	ND365	ND436	ND317	ND365	ND436
0	0.00	0.00	0.00	0.00	0.00	0.00
7	1.46	1.54	1.30	1.70	1.74	1.37
12	3.41	3.31	2.95	2.99	2.85	2.75
17	5.27	5.72	5.01	4.92	3.94	4.70
22	6.09	7.20	6.81	6.58	4.92	6.52
27	7.60	8.08	8.92	7.82	5.73	7.22

Population doublings in control (*N*=3) or TOX2OE-treated (*N*=3) normal donor (ND) CAR T cells stimulated with either CD19, measured over 27 days.

Table S7

Percent	Population	Day	Condition
5.64	PD-1	0	empty
6.15	PD-1	0	TOX2OE
11.9	PD-1	0	empty
8.64	PD-1	0	TOX2OE
7.02	PD-1	0	empty
7.14	PD-1	0	TOX2OE
3.24	PD-1	7	empty
14	PD-1	7	TOX2OE
5.58	PD-1	7	empty
17.5	PD-1	7	TOX2OE
5.92	PD-1	7	empty
19.3	PD-1	7	TOX2OE
1.1	TPEX	0	empty
1.06	TPEX	0	TOX2OE
4.51	TPEX	0	empty
7.9	TPEX	0	TOX2OE
1.8	TPEX	0	empty
3.7	TPEX	0	TOX2OE
8.56	TPEX	7	empty
34.2	TPEX	7	TOX2OE
33.6	TPEX	7	empty
55.2	TPEX	7	TOX2OE
14.9	TPEX	7	empty
43.2	TPEX	7	TOX2OE

Percent of PD-1 and TPEX cells for control (*N*=3) or TOX2OE (N=3) conditions. Each PD-1 line represents the CCR7- PD-1+ population from a separate experiment, measured on the day indicated. Each TPEX line represents the CCR7+ PD-1+ population from those same experiments and days.

Table S8

Condition	Cell type	Name	Size	NES	NOM p-val	FDR q-val
empty	CD4	GSE11057_EFF_MEM_VS_CENT_MEM_CD4_TCELL_UP	174	-1.7590185	0	0
empty	CD4	TCM LOWEST	26	-1.7487444	0.002079	0.00186504
empty	CD4	TEM LOWEST	296	-1.5802127	0	0.0079688
empty	CD4	GSE11057_EFF_MEM_VS_CENT_MEM_CD4_TCELL_DN	169	-1.4665493	0.00930233	0.00930233
empty	CD4	TN LOWEST	727	-1.3255593	0.00287356	0.08089509
empty	CD4	TEM/TN	119	-1.6461123	0.00223714	0.08487143
empty	CD4	TEM HIGHEST	42	-1.2666942	0.12244898	0.09237699
empty	CD4	TEM/TCSM	39	-1.4067495	0.06167401	0.19433945
empty	CD8	EXHAUSTED_DOWN	540	-1.6053562	0	0
empty	CD8	TN LOWEST	727	-1.2995971	0	0.18689534
TOX2OE	CD4	EXHAUSTED_DOWN	544	1.6247214	0	0
TOX2OE	CD4	EXHAUSTED_UP	415	1.3035729	0.0046875	0.00461361
TOX2OE	CD4	TN/TCSM	15	1.2598879	0.20537429	0.1514984
TOX2OE	CD4	TCM/TCSM	11	1.2721548	0.2	0.17624803
TOX2OE	CD4	TN/TEM	66	1.2989137	0.07372401	0.20236231
TOX2OE	CD8	TN/TEM	64	1.7683847	0	0.02286429
TOX2OE	CD8	TN/TCM	32	1.6702145	0.01132686	0.03927499
TOX2OE	CD8	TCM/TEM	156	1.4328537	0.01453104	0.04602858
TOX2OE	CD8	TEM LOWEST	294	1.4683006	0.00365854	0.07517143
TOX2OE	CD8	TN HIGHEST	86	1.2955626	0.09198813	0.08737787
TOX2OE	CD8	EXHAUSTED_UP	403	1.1700506	0.10514019	0.09432048
TOX2OE	CD4	TCM/TEM	11	1.3400471	0.14087301	0.24162996
TOX2OE	CD8	TCSM/TEM	22	1.363053	0.1098546	0.25463882
TOX2OE	CD4	TN/TCM	32	1.4149427	0.07543521	0.29776803
empty	CD8	TCM/TEM	10	-1.2348009	0.2104019	0.33696193
TOX2OE	CD8	TSCM/TN	51	1.1398724	0.25437203	0.39925388
TOX2OE	CD8	TEM/TCM	12	1.1015645	0.32384342	0.40055364
TOX2OE	CD8	TN/TCSM	15	1.0601611	0.38421053	0.4179135
TOX2OE	CD8	TCM/TN	96	1.1549929	0.2189781	0.44951802
TOX2OE	CD8	TEM/TN	117	1.1857833	0.16083916	0.48064393
TOX2OE	CD8	TEM/TCSM	38	0.9691284	0.4976599	0.5145581
empty	CD8	GSE23321_CENTRAL_VS_EFFECTOR_MEMORY_CD8_TCELL_UP	170	-0.9759548	0.525641	0.525641
empty	CD4	TCM/TN	97	-0.975172	0.51502144	0.59559155
empty	CD4	TCSM/TEM	25	-0.9043815	0.61587983	0.62167305
TOX2OE	CD8	TEM HIGHEST	39	0.9077181	0.60392797	0.6780849
empty	CD4	TEM/TCM	12	-0.9967925	0.47186148	0.6907386
empty	CD4	TSCM/TN	52	-1.05242	0.33860046	0.7304032
empty	CD4	TN HIGHEST	84	-0.8457934	0.78494626	0.83125734
empty	CD8	TCM/TCSM	11	-0.7418327	0.80046403	0.8594027
control	CD8	TCM/TN	198	-0.757337	0.98029554	0.9245221
TOX2OE	CD4	TCM/TEM	156	0.6709648	1	0.97686905
TOX2OE	CD8	GSE23321_CENTRAL_VS_EFFECTOR_MEMORY_CD8_TCELL_DN	178	0.6580738	0.9946808	0.9946808
TOX2OE	CD4	TCM/TN	199	0.79350144	0.9320755	1
control	CD8	TCM LOWEST	27	-0.8639023	0.6467662	1

Gene set enrichment analysis (GSEA) results for RNAseq counts from empty vector or TOX2 overexpression samples (N=3 for each group), in both $CD4^+$ and $CD8^+$ T cells. Gene set size, normalized enrichment score (NES), nominal p-value, and false discovery rate (FDR) q-values are reported. FDR q-value < 0.25 was considered significant. Gene sets are referenced in main text.

Table S9
Table is included as an additional excel file
Gene names and differential expression data from DESeq analysis for genes activated or repressed by TOX2, according to RNAseq. For genes listed as "Repressed," the baseMean, log2FoldChange, lfcSE, stat, pvalue, and padj are from a comparison of TOX2 overexpression versus control empty overexpression. For genes listed as "Activated," all columns are from a comparison of TOX2 shRNA knockdown vs. non-targeting shRNA control.

Table S10

Percent	Condition	Donor	Population
33.7	control	ND502	TCM
46.7	TOXKD	ND502	TCM
44.5	control	ND561	TCM
67.4	TOXKD	ND561	TCM
33.2	control	ND569	TCM
55.9	TOXKD	ND569	TCM
56.9	control	ND502	TEM
45.2	TOXKD	ND502	TEM
54.4	control	ND561	TEM
31.9	TOXKD	ND561	TEM
65.5	control	ND569	TEM
43.1	TOXKD	ND569	TEM

Percent of TCM and TEM cells for control (*N*=3) or shTOX(N=3) conditions. Each TCM line represents the CCR7+ CD45RO+ population from a separate experiment. Each TEM line represents the CCR7- CD45RO+ population from those same experiments.
